# Factors that influence the recognition, reporting and resolution of incidents related to medical devices and other healthcare technologies: a systematic review

**DOI:** 10.1186/s13643-015-0028-0

**Published:** 2015-03-29

**Authors:** Julie Polisena, Anna Gagliardi, David Urbach, Tammy Clifford, Michelle Fiander

**Affiliations:** Canadian Agency for Drugs and Technologies in Health, 600-865 Carling Avenue, Ottawa, ON K1S 5S8 Canada; Department of Epidemiology and Community Medicine, Faculty of Medicine, University of Ottawa, 451 Smyth Road, Ottawa, ON K1H 8 M5 Canada; Toronto General Research Institute, University Health Network, 200 Elizabeth Street, Toronto, ON M5G 2C4 Canada; Department of Surgery and Institute of Health Policy, Management and Evaluation, University of Toronto, 200 Elizabeth Street, Toronto, ON M5G 2C4 Canada; Cochrane Effective Practise & Organisation of Care (EPOC) Group, Centre for Practice Changing Research, Ottawa Hospital Research Institute, 501 Smyth Road, Box 711, Ottawa, ON K1H 8 L6 Canada

**Keywords:** Medical device, Healthcare technology, Post-market surveillance, Incidents, Patient safety, Hospital

## Abstract

**Background:**

Medical devices have improved the treatment of many medical conditions. Despite their benefit, the use of devices can lead to unintended incidents, potentially resulting in unnecessary harm, injury or complications to the patient, a complaint, loss or damage. Devices are used in hospitals on a routine basis. Research to date, however, has been primarily limited to describing incidents rates, so the optimal design of a hospital-based surveillance system remains unclear. Our research objectives were twofold: i) to explore factors that influence device-related incident recognition, reporting and resolution and ii) to investigate interventions or strategies to improve the recognition, reporting and resolution of medical device-related incidents.

**Methods:**

We searched the bibliographic databases: MEDLINE, Embase, the Cochrane Central Register of Controlled Trials and PsycINFO database. Grey literature (literature that is not commercially available) was searched for studies on factors that influence incident recognition, reporting and resolution published and interventions or strategies for their improvement from 2003 to 2014. Although we focused on medical devices, other health technologies were eligible for inclusion.

**Results:**

Thirty studies were included in our systematic review, but most studies were concentrated on other health technologies. The study findings indicate that fear of punishment, uncertainty of what should be reported and how incident reports will be used and time constraints to incident reporting are common barriers to incident recognition and reporting. Relevant studies on the resolution of medical errors were not found. Strategies to improve error reporting include the use of an electronic error reporting system, increased training and feedback to frontline clinicians about the reported error.

**Conclusions:**

The available evidence on factors influencing medical device-related incident recognition, reporting and resolution by healthcare professionals can inform data collection and analysis in future studies. Since evidence gaps on medical device-related incidents exist, telephone interviews with frontline clinicians will be conducted to solicit information about their experiences with medical devices and suggested strategies for device surveillance improvement in a hospital context. Further research also should investigate the impact of human, system, organizational and education factors on the development and implementation of local medical device surveillance systems.

**Electronic supplementary material:**

The online version of this article (doi:10.1186/s13643-015-0028-0) contains supplementary material, which is available to authorized users.

## Background

The US Food and Drug Administration (FDA) defines a medical device as an instrument used to diagnose, treat or prevent a disease or abnormal physical condition without any chemical action in the body [1]. Devices have improved care delivery and associated outcomes for many conditions. Despite their benefit, an audit of the UK National Patient Safety Agency over 7 months found that 1,021 of 12,084 patient safety incidents were due to devices. Although the reports lacked details about the device, procedure, outcomes and factors causing the incident, the audit also found that device-related incidents were caused by device failure (43.8%), inappropriate use (29.3%), lack of training (12.3%) and inadequate maintenance (1.5%) [2].

Lawton *et al*. developed a ‘contributory factors framework’ from the published literature on factors associated with patient safety incidents in a hospital context. The authors found that two main contributory factors related to patient safety incidents were active failures (that is, any failure in performance by the end-user) and individual factors (that is, characteristics of the persona delivering the case that may contribute in some way to active failures) [3]. In addition, Pfeiffer *et al*. proposed a framework on barriers and motivators for incident reporting. They concluded that individual, organizational and incident reporting systems factors impacted reporting behaviour [4]. While not specific to devices, a systematic review identified 1,676 factors contributing to patient safety incidents in 83 eligible studies and categorized factors into 20 domains including active failure in performance or behaviour, clinician, team, institution, system, culture, training, accountability and patient factors [5].

Widespread concern about device-related incidents has prompted numerous calls for enhanced monitoring. Medical devices are used in hospitals on a routine basis, and prospective surveillance would more closely monitor and identify incidents closer to real time. Post-market surveillance (PMS), therefore, represents a crucial approach to prevent and mitigate potential harm associated with the use of devices. In addition to safety and effectiveness, PMS would facilitate the collection of incident data, guide the development of training and policies to improve patient safety and influence decisions on the purchase and replacement of medical devices.

Research to date has been largely limited to describing incident rates, so the optimal design of a hospital-based surveillance system remains unclear [2,5-9]. Given limited guidance on how to develop and implement a hospital-based PMS system, a 1-day meeting was convened in 2011 involving researchers, clinicians, surgeons, government regulators, industry and patient advocacy groups from Canada and the US to identify specific gaps in knowledge and prioritize ongoing research [10]. Key recommendations from the 2011 meeting were the need for further research to explore the nature of hospital-based PMS systems and the factors that influence the reporting of incidents at the hospital level [10]. For a holistic perspective, the authors decided that the recognition and resolution of medical device-related incidents also were important considerations for PMS systems.

Our study objectives were twofold: i) to explore factors that influence the recognition, reporting and resolution of incidents by healthcare professionals related to the use of medical devices in hospitalized patients and ii) to investigate interventions or strategies used by health professionals intended to improve the recognition, reporting and resolution of medical device-related incidents in hospitalized patients. For our study, resolution was defined as interventions use to reduce the risk of similar incidents from reoccurring.

## Methods

### Approach

A traditional systematic review of the medical and grey literature (literature that is not commercially available) was conducted to identify and describe studies on factors that influence the recognition, reporting and recognition of the incidents in a hospital. A protocol for the review was written *a priori* and was followed in detail. We identified, categorized and analysed the factors into various themes, and the results are presented according to the Preferred Reporting Items for Systematic Reviews and Meta-Analyses (PRISMA) reporting guidelines [11].

### Literature search strategy

Peer-reviewed literature searches were conducted for our systematic review. The following bibliographic databases were searched: MEDLINE (1996-), Embase (1980-), the Cochrane Central Register of Controlled Trials and PsycINFO database. The search strategy consisted of both controlled vocabulary, such as the National Library of Medicine’s MeSH (Medical Subject Headings), and keywords. The main search concepts were medical errors, post-marketing product surveillance, medical device recalls and safety-based medical device withdrawals combined with prostheses and implants, adverse events and medical errors. Grey literature was identified by searching relevant sections of the Grey Matters checklist [12]. Upon preliminary searches, an insufficient amount of evidence specific to medical devices was identified, so the search was expanded to include factors that influence the recognition, reporting and resolution of errors related to the use of other healthcare technologies that may be relevant to medical device-related incidents. Searches were conducted until July 31, 2014 (see Additional file 1: Table S1).

### Selection criteria

The selection criteria included empirical quantitative and qualitative studies published from 2003 to 2014 that identified factors associated with incidents in a hospital setting. Although our primary focus was medical device-related incidents, additional health technologies, such as drug therapies, diagnostic and screening tests, vaccines and surgical and non-invasive procedures, were eligible for inclusion. The literature was restricted to studies published in languages spoken by the authors, including English, French, Italian and Spanish.

Studies were not eligible if they reported the nature or frequency of adverse events or incidents associated with the use of a healthcare technology without examining factors that influence their recognition or reporting or how they were addressed were excluded. Studies that involved primarily trainees, such as medical students, interns or residents, were not relevant to this review. Ineligible articles also included studies on automated, regional or national surveillance systems using administrative data or medical records, automated adverse event or incident reporting by the healthcare technology itself and advisories, warnings or recalls by manufacturers or regulators. Finally, articles in the form of abstracts, letters, commentaries, newsletter articles or editorials also were excluded.

### Article selection

The principal investigator and a research assistant independently reviewed the titles and abstracts of search results and selected articles for inclusion based on the eligibility criteria. Rather than resolving selection differences, all those selected by at least one reviewer were retrieved since ultimate judgment about inclusion must often be reserved until the full text was examined.

### Data extraction

A data extraction form was developed to collect information on study design and findings including factors, such as device features, failure or malfunction, clinician or institutional characteristics, system or patient factors, and types and severity of adverse event. The principal investigator extracted data from all eligible studies, and data were reviewed independently by a research assistant. Any disagreements were discussed until a consensus was reached.

### Quality assessment

The instrument used to assess the included studies varied by their design. For descriptive studies, the appropriateness of the research design, recruitment strategy and data collection, potential researcher bias, ethical considerations, data analysis and reporting of study findings was reviewed for each individual study [13]. For comparative studies, the SIGN50 checklists for cohort and case-control were applied [14,15]. In general, the studies were assessed on their appropriateness of design to answer the research questions, potential risk of biases and confounding, as well as relevance of findings to our scope. The principle investigator wrote the comments for each question in the quality assessment tool for individual studies, and a research assistant verified the responses. The strengths and limitations of the individual studies were described.

### Data analysis

Data were tabulated, and findings were examined to discuss the quantity, design and quality of studies. The factors contributing to incident recognition, reporting and resolution and interventions or strategies to improve medical device surveillance in a hospital context were described.

## Results

### Quantity of research available

**Figure 1:**
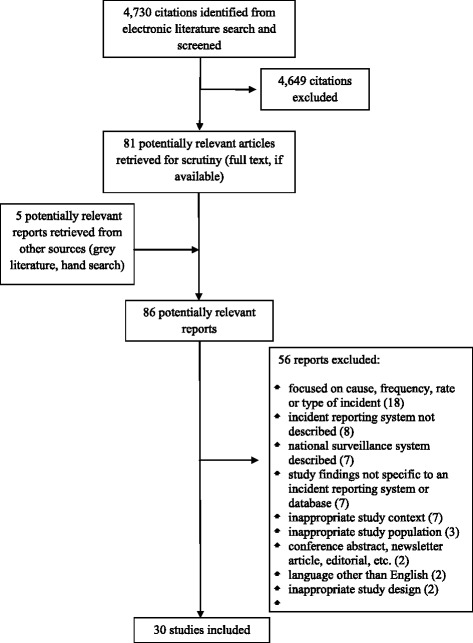
**PRISMA flowchart.**

The literature search identified 4,730 citations. From these, 81 potentially relevant full-text articles were retrieved for further scrutiny and five potential articles were identified through hand-searching. Thirty studies were selected for inclusion. The PRISMA flowchart in Figure 1 details the process of study selection.

### Study characteristics

The publication years ranged from 2004 to 2013. Nine studies were published in the US [16-24], five in the UK [25-29], four in Australia [30-33], three in Canada [34-36], two each in Italy [37,38] and Korea [39,40] and one each in Turkey [41], China [42], Pakistan [43], France [44] and the Netherlands [45]. Two studies examined incidents associated with the medical equipment or devices [42,44], and one Canadian study investigated the barriers and facilitators to medication error reporting in four community hospitals [34]. The remaining studies did not focus on incidents related to the use of any specific healthcare technologies. Table 1 presents the main purpose of selection studies and their corresponding frequency. Half of the selected studies (*n* = 15) focused on the attitudes towards, barriers to, experience in, facilitators to and/or perceptions of adverse event, error and incident reporting. Most studies were surveys (*n* = 15) [16,18,19,22,24,27,30,33,35,37,38,40,41,43,44], six were interviews [21,26,28,29,36,39] and three each were descriptive [20,25,42] and involved focus groups [17,23,32]. Other studies were a review of patient records [45], non-equivalent clinical trial [31] and a mix methodology of interviews and focus groups [34]. The complete study characteristics of the included studies are outlined in Table 2.

**Table 1:** **Frequency of main purpose of included studies**

**Main purpose**	**Number of studies**
Attitudes towards, barriers to, experience in, facilitators to and/or perceptions of adverse event, error and incident reporting	15 [15,18,20,22,23,26-29,31,32,35,37,38,42]
Description of causes and health consequences and/or effectiveness of prevention strategies for adverse events	3 [19,21,44]
Hospital staff’s attitude towards, knowledge of and/or behaviour in medical errors	2 [36,43]
Perception of patient safety culture among hospital staff	2 [39,40]
Error recovery strategies by healthcare providers	2 [16,34]
Impact of incident reporting system in surgery	1 [24]
Perceived effectiveness of incident reporting in mental health and acute care hospitals	1 [25]
Effectiveness of real and potential medical errors on healthcare providers	1 [17]
Effectiveness of improvement incident reporting strategies	1 [30]

**Table 2:** **Study characteristics of included studies**

**First author; country; year**	**Number of centres; number of participants (errors); sponsor**	**Study objective(s) (verbatim)**	**Study design and duration; clinical category(ies)**
Wong *et al*. [25]; UK; 2013	1 ophthalmic facility; 579 incidents; no funding sources	To examine the impact of patient safety incident reporting on errors during vitreoretinal surgery	Descriptive; January 1997 to December 2009; vitreoretinal surgery
Anderson *et al.* [26]; UK; 2012	2 large, teaching hospitals; 62 healthcare practitioners (for example, doctors, nurses and managers); government	To examine the perceived effectiveness of incident reporting in improving safety in mental health and acute hospital settings	Documentary analysis and semi-structured interviews; NR; mental health and acute care
Flotta *et al*. [37]; Italy; 2012	Hospitals across 20 Italian regions; 696 physicians; none	To investigate physicians’ knowledge about evidence-based patient safety practices, their attitudes on preventing and managing medical errors and to explore physicians’ behaviour when facing medical errors	Survey; NR; general medicine, general surgery, medical specialities, ICU/ED
Hartnell *et al*. [34]; Canada; 2012	4 community hospitals; 30 participants (pharmacists, physicians, nurses); government	1. To identify incentives barriers and facilitators to encourage medication error reporting as perceived by front-line hospital staff	Key informant interviews and focus groups; NR; NR
		2. To understand why certain factors serve as barriers	
		3. To explore how some hospitals have successfully removed barriers	
Heard *et al*. [30]; Australia; 2012	The Australian and New Zealand College of Anaesthetists; 327 consultant anaesthesiologists and 103 anaesthesia residents, NR	To explore the attitudes and barriers of anaesthesiologists to reporting adverse events and errors	Anonymous, self-administered survey; NR; anesthesiology
Hwang *et al*. [39]; Korea; 2012	42 general hospitals; 42 nurses; government	To explore the barriers to and factors facilitating the operation of patient safety incident reporting systems	Interviews and emails; July 2010 to April 2011; NR
Albolino *et al*. [38]; Italy; 2010	14 hospitals; 820 healthcare workers; government	To assess workers’ experience of patient safety incidents and their expectations on incident reporting	Written survey; April/May 2006 to January 2007; surgery, medicine, obstetrics and gynaecology, intensive care, radiology and laboratory, rehabilitation and other
Bodur and Filiz [41]; Turkey; 2010	1 general hospital, 1 teaching hospital, and 1 university hospital; 309 participants (physicians and nurses); NR	1. To determine the validity and reliability of the Hospital Survey on Patient Safety Culture	Cross-sectional survey; not specified
		2. To evaluate physicians’ and nurses’ perceptions of patient safety in Turkish hospitals	
		3. To compare the findings with US hospital settings	
Chien *et al*. [42]; China; 2010	1 2,300-bed university hospital; NR; NR	To present information framework to build and to enhance the CED on the medical equipment management capabilities with an example for portable physiological monitors used in nursing department	Descriptive; NR; NR
Espin *et al*. [36]; Canada; 2010	3 hospitals (1 urban academic tertiary hospital, 1 community hospital, 1 academic paediatric hospital); 37 nurses; government and academic	To explore emergent factors influencing nurse’ error reporting preferences, scenarios were developed to probe reporting situations in the ICU	Semi-structured interviews; NR; ICU
Henneman *et al*. [17]; US; 2010	2 urban university medical centres and 2 community hospitals; 20 nurses; non-profit organization	To describe error-recovery strategies used by critical care nurses	Focus groups; NR; critical care units
Loren *et al*. [16]; US; 2010	NR; 1,673 healthcare facility-based risk managers; government and academic	To conduct a national survey of risk managers’ attitudes regarding patient safety and error disclosure and to compare the results with a previously published survey of medical physicians	Survey; November 2004 to March 2005; NR
Malik *et al*. [43]; Pakistan; 2010	600- bed tertiary care facility; 114 doctors 103 and nurses; NR	To determine the attitudes and perceived barriers towards incident reporting tertiary care health professionals in Pakistan	Survey; NR; medicine (non-surgical), ICU, surgery, anaesthesia, gynaecology and obstetrics, paediatrics, ER and others
Smits *et al*. [45]; Netherlands; 2010	21 hospitals (4 university, 6 tertiary teaching, and 11 hospitals); 744 AEs identified in 7,926 patient records and 55 physicians reviewed patient records; government	To gain more insight into	Retrospective patient record review; August 2005 to October 2006; excluded admissions of psychiatry, obstetrics and children <1 year old
		1. The causes of AEs	
		2. The relationship between the causes of AEs and the preventability and health consequences of the AEs	
		3. Potential prevention strategies to prevent AEs and	
		4. The relevance of the prevention strategies for each main causal factor type	
Kreckler *et al*. [27]; UK; 2009	General surgical department in teaching hospital; 55 doctors and 82 nurses; NR	To evaluate the process of incident reporting in a surgical setting. In particular, the influence of event outcome on reporting behaviour; staff perception of surgical complications as reportable events	Anonymous web-based questionnaire survey; January to March 2007; general surgery
Hohenhaus [24]; US; 2008	2 US states; 173 nurses; government	To evaluate current practice of reporting medical error among nurses in the emergency department	Survey; April to June 2005; emergency medicine
Kroll *et al*. [28]; UK; 2008	10 hospitals; 38 junior doctors; none	To investigate experiences of and responses to medical error amongst junior doctors and to examine challenges junior doctors face and the support they receive	Semi-structured interviews; NR; NR
Bognár *et al*. [18]; US; 2007	3 academic hospitals; 61 PCS team members; non-profit organization	To explore the impact of real and potential medical errors on PCS team members	Survey; NR; paediatric cardiac surgery
Cooke *et al*. [35]; Canada; 2007	1 academic cancer care centre; 125 radiotherapists, nurses, dosimetrists, doctors and other staff	To motivate improvements in an organizational system by measuring staff perceptions of the organization’s ability to learn from incidents and by analysing their personal experience of incidents	Survey, NR; oncology
Evans *et al*. [31]; Australia; 2007	2 regional hospitals; 14 doctors and 19 nurses; government	To assess the effectiveness of an intervention package comprising intense education, a range of reporting options, changes in report management and enhanced feedback, in order to improve incident-reporting rates and change the types of incidents reported	Non-equivalent group controlled clinical trial (ten intervention and ten control units); June to August 2003; medical units, surgical units, ICUs, EDs, neurology, cardiology and gastrointestinal surgery
Kim *et al*. [40]; Korea; 2007	8 university hospitals; 886 nurses; government	1. To describe the frequency of error reporting for near misses and harmless but potentially harmful errors	Survey; NR; internal medicine, ICU, surgical unit, ER, OR, obstetrics unit and other
		2. To describe nurses’ perceptions of patient safety culture in their working unit and hospital, their supervisors’ attitudes towards patient safety issues, communication channels, and processes regarding patient safety	
		3. To examine whether nurses’s perceptions were significantly associated with their work experience, work position, type of unit, age and working hours	
Evans *et al*. [33]; Australia; 2006	3 principle referral hospitals, 1 major referral hospital, and two major rural base hospitals; 773 participants (physicians and nurses); NR	To investigate by profession	Cross sectional survey; November 2001 and June 2003; NR
		1. Awareness and use of current incident reporting system	
		2. The types of incidents staff are more likely to report and believe should be reported	
		3. The barriers to reporting	
Schectman and Plews-Ogan [19]; US; 2006	1 academic medical centre; 120 physicians; NR	To assess the safety reporting behaviour and witnessed AEs or near misses	Anonymous survey; spring 2005; internal medicine
Ursprung *et al*. [20]; US; 2005	20-bed tertiary care medical-surgical NICU; 338 errors; government	To conduct a pilot study to determine the feasibility (whether audits were completed each day they were attempted and whether staff disclosed errors during routine daily work) and utility (whether the safety questions audited detected important errors) of 36-item real-time safety auditing during routine clinical work in the ICU	Descriptive; 28 January to 4 March 2003; NICU
Cohen *et al*. [22]; US; 2004	489-bed non-teaching suburban community hospital; NR; NR	To determine comprehensive patient safety programme’s impact on two specific putative measures of the safety culture: event-reporting rates and surveys of staff opinion	Survey; January 2000 to March 2003 in three phases; NR
Demiris *et al*. [21]; US; 2004	8 rural hospitals in Missouri; 30 participants (administrators, physicians and nurses); NR	1. To investigate rural healthcare providers’ and administrators’ attitudes towards patient safety and their attitudes towards and expectations of an adverse event reporting system	Interviews; NR; NR
		2. To provide insight into the organizational culture and level of readiness as well as to identify critical issues pertaining to the rural context that needs to inform the design of such strategies	
Jeffe *et al*. [23]; US; 2004	20 academic and community hospitals; 49 staff nurses, 10 nurse managers, 30 physicians; government	To gain insight into workers’ perspectives about key concepts and issues regarding medical error reporting in hospitals	Focus groups; May to June 2002; NR
Kingston *et al*. [32]; Australia; 2004	5 units across 3 tertiary metropolitan public hospitals; 33 participants (medical and nursing staff; NR	1. To examine attitudes of medical and nursing staff towards reporting incidents	Focus groups; March 21 to 22, 2002; NR
		2. To identify measures to facilitate incident reporting	
Mazeau *et al*. [44]; France; 2004	2 hospitals; 216 participants (physicians paid on hourly basis, head nurses, nurses, other caregivers, and administrative personnel); NR	1. To evaluate staff knowledge of hospital medical device surveillances and to describe potential determining factors of this knowledge	Cross-sectional survey; 3 December 2001 to 15 January 2002; NR
		2. To design a method suitable for any evaluation of hospital staff knowledge about what must be indisputably known by a large part of the staff	
Waring [29]; UK; 2004	1 medium-sized district general hospital; 28 interviews with 3 senior medical representatives and 25 specialist physicians; NR	The attitudes of medical physicians towards adverse incident reporting in health care, with particular focus on the inhibiting factors or barriers to participation are explored	Interviews; 2001 to 2003; anaesthesia, acute medicine, obstetrics, rehabilitation and surgery

AE, adverse event; AMDE, adverse medical device event; ED, emergency department; ER, emergency department; ICD, International Classification of Diseases; ICU, intensive care unit; NA, not applicable; NICU, neonatal intensive care unit; NR, not reported; OR, operating room; PCS, paediatric surgical team; UK, United Kingdom.

### Quality assessment of included studies

The methodological quality of the selected studies was moderate. Where applicable, a verbal or signed informed participant consent was obtained for most studies [21,34,41,46,47]. Numerous studies described how participants were selected randomly to obtain various perspectives from healthcare providers [21,34,41,44,46]. None of the studies reported any potential biases as a result of the interactions and relationship between the researchers and participants. Although many studies described the statistical analyses where appropriate and presented them in detail, most did not discuss in great detail the contribution of study findings in relation to current practice or policy.

### Summary of findings

Details on the study findings are found in Additional file 2: Table S2.

#### Factors that influence the recognition of incidents by healthcare professionals

In a study on the local medical equipment management system, attributes of manufactured products and their composed parts influenced the occurrence and recognition of device incidents reports [42].

#### Factors that influence the reporting of incidents by healthcare professionals

Hospital staff awareness of a local surveillance or reporting system seemed to vary across six studies [17,19,22,27,33,44]. Although a greater proportion of nurses knew the hospital surveillance process compared with physicians in two surveys [17,44], a similar proportion of doctors and/or nurses correctly identified the process in two other studies [27,33]. Amongst 120 internists surveyed in the US, 41% were not familiar with the safety process at their institution, but 33% knew how to report an adverse event or a near miss [19]. In an Australian survey, over 50% of doctors felt that the incident form was too time-consuming to complete or that the incident was too trivial to report *versus* over 40% of nurses, who felt the same way [33]. One survey of 30 healthcare providers and administrators in eight rural hospitals expressed challenges associated with the training time and implementation and maintenance costs of an electronic reporting system [21]. Based on results of a survey, nurses also were three times as likely to report no-harm events compared with doctors. Factors that would impact the likelihood of reporting an adverse event for both nurses and doctors were level of harm, incident type and profession [27].

A study set in three public hospitals in Turkey found that at least 30% of hospital staff felt that the feedback on and communication about medical errors was open; received feedback informing staff about changes to practice or procedure based on reported errors; were informed of errors that occur in their hospital units; were encouraged to discuss strategies to prevent future errors and were comfortable in ‘speaking up’ if they saw something that may negatively affect resident care. In the same survey, 47% of respondents were afraid to ask questions if something did not seem right. [41] A survey conducted in six hospitals in Australia indicated that over half of doctors and nurses felt that they did not receive any feedback following error reporting, nor were they able to determine if the reports led to actions or changes [33].

Personal attitudes of healthcare professionals towards incident reporting were presented in 16 studies [16,22-24,28-30,32-36,38,39,41,43]. Respondents in a Canadian study performed in four community hospitals listed patient and provider protection and professional compliance as incentives to report medical errors within their facilities [34]. Other incentives identified were to obtain immediate help for the patient, to learn from mistakes and to develop a system to minimize repetition of incidents [43]. Respondents in three surveys found that their organizational culture, in general, supported error reporting and did not think that there was a culture of blame [17,21,41].

Reasons cited in numerous studies for not reporting errors include lack of awareness of what and how to report, fear of repercussion and punishment, mistrust and lack of confidentiality, organizational support, time and easy systems for reporting and follow-up [23,24,33,36,38,39,43]. Fear of blame, rejection of bureaucracy and managerial scrutiny, administrative sanctions, legal penalties and/or perception that incident reporting does not improve patient safety, lack of organizational support and lack of knowledge on incident reporting system and what constitutes an error were other reasons why healthcare professionals did not always report errors [16,28-30,32,35,38,39,41,43].

#### Factors that influence the resolution of incidents by healthcare professionals

We did not identify any relevant studies on factors that influence the resolution of incidents by healthcare professionals. For our study, a resolution is defined as an intervention to reduce the risk of medical device-related incidents from occurring in the future.

#### Interventions or strategies that are meant to improve the recognition, reporting and resolution of incidents by healthcare professionals

Ten studies reported on interventions or strategies intended to enhance the recognition, reporting and resolution of incidents by healthcare professionals [19,20,23,25,26,30,31,37,39,43,45]. An anonymous survey revealed that 75% of anaesthesiologists in Australia agreed or strongly agreed that feedback, role models, legislated protection, ability to report anonymously and clear guidelines are effective strategies to improve adverse event reporting [30]. Other potential strategies include continuous monitoring of data and assessments of a health professional’s performance, evaluations of behaviours regarding safety and safety audits during clinical routine work training [20,45].

In two regional hospitals in Australia, education, various reporting options, change in report management and enhanced feedback showed a significant improvement in reporting rates for both nurses and doctors across numerous departments [31]. In another study, the most frequently measures reported among 42 nurses were the introduction of a reward system, improvement of reporting system, recruitment more staff for patient safety incident management, enhancement of safety culture and education and training opportunities [39]. Two studies indicated that an electronic and/or anonymous system likely would increase incident reporting among hospital staff [19,21]. Additional facilitators to error reporting include clear guidelines, clarification of reporting mechanisms and training of healthcare providers, non-accusatory environment, anonymous reporting mechanisms, sufficient personnel and efficient reporting tools and routine follow-up of reported errors [23].

In terms of error recognition or resolution, participants in focus groups in critical care nursing indicated that knowing all aspects of the patient, other patients in the unit, the plan of care, and referring to critical care policies and procedures, hospital accreditation and unit-based and other standards as examples of effective strategies to identify or correct errors [17]. The majority of respondents in an Italian survey agreed to discuss with colleagues about medical errors, increase information seeking to reduce recurrence of medical errors and report medical errors to their institution to improve the quality of care [37].

## Discussion

Our study identified 30 studies on factors that influence whether and how incidents are recognized and reported by hospital staff and interventions and strategies to improve their recognition, reporting and resolution. One study in our systematic review discussed the recognition of incidents by healthcare professionals in a hospital facility. Furthermore, the central themes identified for the reporting of incidents are personal attitudes, awareness and perception of incident reporting systems, organizational culture and feedback to healthcare professionals (Table 3). Although we were unable to identify relevant studies on the resolution of incidents in a hospital context, participants in the selected studies indicated that they would report errors more frequently if reporting were easier, they were educated about what to report and how, and received timely feedback on actions taken based on reported data. Information sharing with colleagues, knowledge about the patient’s condition and an understanding of hospital policies and procedures were cited as effective strategies to help reduce the risk of similar incidents from reoccurring.

**Table 3:** **Factors influencing the recognition, reporting and resolution of medical device-related incidents**

**Factor**	**First number of studies**
Recognition	
Health technology features	1 study [29]
Reporting	
Personal attitudes of healthcare professionals	16 studies [16,22-24,28-30,32-36,38,39,41,43]
Organizational culture	4 studies [17,21,40,41]
Awareness of incident reporting system	6 studies [17,19,22,27,33,44]
Perception of incident reporting system	5 studies [16,17,21,27,33]
Incentives to incident reporting	2 studies [34,43]
Feedback to healthcare professionals	2 studies [33,41]
Resolution	
None of the studies reported on the resolution of medical device-related incidents	

A systematic review on institutional medical incident reporting systems found that reporting alone is insufficient to reduce the risk of medical errors in a hospital. The authors of the systematic review suggested that successful management of medical risk in hospital facilities occurs in the following three phases: i) risk identification by reviewing the reporting systems and incident and near-misses reports; ii) risk analysis through root cause analysis; and iii) risk control by implementing system changes and improvements [48]. To increase the patient safety of medical device use in a hospital, Hinrichs *et al*. recommend a holistic system among the stakeholders who are responsible for their purchasing process. Their study findings based on observational work, participatory workshop and semi-structured qualitative interviews in five UK hospitals found that decisions among healthcare stakeholders typically are made in isolation across the hospitals. The authors concluded that this occurrence would result in knowledge and training gaps and would have a negative impact on patient care [49].

Our study is not without limitations. We were unable to identify relevant literature on factors that influence the resolution of medical device-related incidents. Although the grey literature was searched to ensure the comprehensiveness of reports, these studies represent a dearth of evidence in this area. Although the literature was restricted to studies published in languages spoken by the authors, Morrison *et al*. found no systematic bias when English-language restrictions were imposed in systematic reviews [50]. Most studies in our review were not specific to medical device-related incidents, but some of the findings can be extrapolated to medical device surveillance systems or incident reporting databases. The type of studies in this review precluded us from investigating which factors aforementioned have the great influence on the recognition, reporting and resolution of incidents related to the use of healthcare technologies. We feel that it is important to highlight the insufficient volume of quality evidence related to medical devices and other healthcare technologies that can lead to enhanced patient safety in a hospital facility.

Our systematic review is the first instalment of a two-part research project on the safety of medical device use in hospitals to inform policy for effective post-market surveillance. As more information related to the use of medical devices is needed, the next instalment involves telephone interviews with general, orthopaedic and vascular surgeons, cardiologists and interventional radiologists and nurses in two teaching Canadian hospitals. The interviews will explore factors that influence medical device-related incident recognition, reporting and resolution; awareness of warnings or recalls and of evidence on safety and effectiveness for devices; and whether and how they inform their patients of device-related risks. The same interviews also will solicit information about the nature of their hospital-based device incident reporting systems and suggestions for improvement. The results of this systematic review will help to guide the interview questions. To contribute to the design and development of a medical device surveillance system, an investigation of other sectors that involve the design, development and use of a technology, such as home appliance, transport and machinery and equipment, to understand their mechanisms in place to recognize, report, resolve and reduce the risk of errors and malfunctions associated with its use may be warranted. This exercise also would incorporate the feasibility and appropriateness of these mechanisms to a device surveillance system in a hospital facility.

## Conclusions

Our systematic review included 30 studies that describe factors that influence the recognition and reporting of incidents in a hospital setting were included. Findings in these studies suggest four main barriers to error reporting as follows: fear of punishment or censure, uncertainty regarding what should be reported, uncertainty as to how incident reports will be used and lack of time. Potential strategies to improve incident reporting include accessible electronic error reporting systems, training about what to report and how and feedback on actions taken based on error reported. The quality of evidence, however, is insufficient. Further research will involve interviews with surgeons and registered nurses to inquire about their experiences with medical device-related incidents in a hospital facility.

## Additional files

Additional file 1: Table S1. Literature search strategy. The additional file describes the literature search strategy. Additional file 2: Table S2. Study findings on factors that influence the recognition, reporting and resolution of incidents by healthcare professionals. The additional file presents the study findings on factors that influence the recognition, reporting and resolution of incidents by health care professionals.
