# Supplementary material for: Factors that influence the recognition, reporting and resolution of incidents related to medical devices and other healthcare technologies: a systematic review
Source: Syst Rev. 2015 Mar 29;4:37. doi: 10.1186/s13643-015-0028-0 (PMC4384231; doi:10.1186/s13643-015-0028-0)
Supplement: Additional file 1: Table S1. — Literature search strategy. The additional file describes the literature search strategy. [file 13643_2015_28_MOESM1_ESM.docx]

Additional file 1: Table S1. Literature search strategy.

| **OVERVIEW** | |  | |
| --- | --- | --- | --- |
| Interface: | | OvidSP | |
| Databases: | | Embase <1980 to 2013 Week 52>  Ovid Medline <1996 to Week 52 2013>  Ovid Medline In-Process & Other Non-Indexed Citations < December 31, 2013>  **Note:** Subject headings have been customized for each database. Duplicates between databases were removed in Reference Manager. | |
| Date of Search: | | December 31, 2013 | |
| Alerts: | | Search updates began April 14, 2013 and are ongoing at monthly intervals. | |
| Study Types: | | Systematic reviews; meta-analyses; technology assessments; randomized controlled trials; controlled clinical trials; multicenter studies; cohort studies; cross-over studies; case control studies; comparative studies. | |
| Limits: | | Humans | |
| **SYNTAX GUIDE** | | |  |
| / | At the end of a phrase, searches the phrase as a subject heading | | |
| .sh | At the end of a phrase, searches the phrase as a subject heading | | |
| MeSH | Medical Subject Heading | | |
| fs | Floating subheading | | |
| exp | Explode a subject heading | | |
| * | Before a word, indicates that the marked subject heading is a primary topic;  or, after a word, a truncation symbol (wildcard) to retrieve plurals or varying endings | | |
| # | Truncation symbol for one character | | |
| ? | Truncation symbol for one or no characters only | | |
| ADJ | Requires words are adjacent to each other (in any order) | | |
| ADJ# | Adjacency within # number of words (in any order) | | |
| .ti | Title | | |
| .ab | Abstract | | |
| .hw | Heading Word; usually includes subject headings and controlled vocabulary | | |
| .pt | Publication type | | |
|  |  | | |

| **Medline Strategy** |
| --- |

1 *Medical Errors/ (8708)

2 incident? reporting.ti. (269)

3 exp food/ (1077970)

4 p?ediatric$.ti,ab,hw. or (child$ or neonat$ or neo-nat$ or infant? or perinat$ or peri-natal$ or "in vivo" or "in vitro").ti,ab,hw,pt. (4032684)

5 exp Child/ (1568825)

6 (aa or dt).fs. (2238128)

7 placebo?.ti,ab,hw. (185191)

8 drug safety.ti,ab. (2638)

9 ((safety and efficacy) or (safety and effectiveness) or treatment or versus).ti. (982749)

10 or/3-9 [Concepts to exclude] (7203868)

11 (equipment failure/ or equipment failure analysis/ or prosthesis failure/ or equipment safety/) and ((new or newer or newly) adj2 (device or devices or product?)).ti,ab. (534)

12 (equipment failure/ or equipment failure analysis/ or prosthesis failure/ or equipment safety/) and surveillance.ti,hw. (738)

13 (equipment design/ or orthodontic appliance design/ or prosthesis design/) and (Safety Management/ or Risk Management/) (468)

14 "Equipment and Supplies"/ and medical errors/ (53)

15 (Failure adj4 ((new or newer or newly or medical) adj2 (device or devices or equipment or product?))).ti,ab. (95)

16 (device? failure or equipment failure or product? failure).ti,ab. (912)

17 Product Surveillance, Postmarketing/ and ae.fs. (3184)

18 (exp "prostheses and implants"/ or stents/ or drug-eluting stents/ or suburethral slings/ or suture anchors/ or urinary sphincter, artificial/ or visual prosthesis/ or defibrillators, implantable/ or exp electrodes, implanted/ or exp pacemaker, artificial/ or cardiac resynchronization therapy devices/ or exp catheters/ or infusion pumps/ or infusion pumps, implantable/) and (surveillance and (device or devices or product? or post-market$ postmarket$ or market$)).ti,ab. (389)

19 (exp "prostheses and implants"/ or stents/ or drug-eluting stents/ or suburethral slings/ or suture anchors/ or urinary sphincter, artificial/ or visual prosthesis/ or defibrillators, implantable/ or exp electrodes, implanted/ or exp pacemaker, artificial/ or cardiac resynchronization therapy devices/ or exp catheters/ or infusion pumps/ or infusion pumps, implantable/) and medical errors/ (321)

20 (exp "prostheses and implants"/ or stents/ or drug-eluting stents/ or suburethral slings/ or suture anchors/ or urinary sphincter, artificial/ or visual prosthesis/ or defibrillators, implantable/ or exp electrodes, implanted/ or exp pacemaker, artificial/ or cardiac resynchronization therapy devices/ or exp catheters/ or infusion pumps/ or infusion pumps, implantable/) and (regulatory or government? or policy mak$ or decision mak? or (regulation? adj3 (device? or product?))).ti. (137)

21 ((medical device? or medical product?) and (surveillance or monitoring or reporting or detecting or safety or postmarket$ or post-market$ or after market)).ti. (284)

22 ((medical device? or medical product?) adj2 adverse event?).ti,ab. (34)

23 (exp "prostheses and implants"/ae or stents/ae or drug-eluting stents/ae or suburethral slings/ae or suture anchors/ae or urinary sphincter, artificial/ae or visual prosthesis/ae or defibrillators, implantable/ae or exp electrodes, implanted/ae or exp pacemaker, artificial/ae or cardiac resynchronization therapy devices/ae or exp catheters/ae or infusion pumps/ae or infusion pumps, implantable/ae) and ((reporting or detection or detecting or identifying).ti. or ((report$ or detect$ or identif$ or tracking or track?) adj3 (system? or process or processes or model? or guideline?)).ab.) (546)

24 (exp "prostheses and implants"/ or stents/ or drug-eluting stents/ or suburethral slings/ or suture anchors/ or urinary sphincter, artificial/ or visual prosthesis/ or defibrillators, implantable/ or exp electrodes, implanted/ or exp pacemaker, artificial/ or cardiac resynchronization therapy devices/ or exp catheters/ or infusion pumps/ or infusion pumps, implantable/) and Consumer Product Safety/ (147)

25 Consumer Product Safety/ and MEdical errors/ (10)

26 (Government Regulation/ and medical device?.ti.) or device regulation?.ti. (137)

27 "product recalls and withdrawals"/ or medical device recalls/ or safety-based medical device withdrawals/ (188)

28 (or/11-27) not 10 [Set 1] (5108)

29 Product Surveillance, Postmarketing/ (5763)

30 29 not (or/3-5,7-8,28) [Set 2] (2465)

31 (medical device? or medical product?).ti,ab. (8890)

32 exp Quality Assurance, Health Care/ or Quality of health care/ (309183)

33 (and/31-32) not (or/10,28,30) [Set 3] (452)

34 (randomized controlled trial or controlled clinical trial).pt. or randomized.ab. or placebo.ab. or clinical trials as topic.sh. or randomly.ab. or trial.ti. (946983)

35 exp animals/ not humans.sh. (4091851)

36 34 not 35 [Cochrane RCT Filter 6.4.d Sens/Precision Maximizing] (875433)

37 multicenter study/ or clinical trial/ (641413)

38 ("research support american recovery and reinvestment act" or research support nih extramural or research support nih intramural or research support non us govt or research support us govt non phs or research support us govt phs).pt. (7622594)

39 congresses.pt. (61515)

40 comparative study/ or evaluation studies/ or retrospective studies/ (2281040)

41 case reports.pt. (1698354)

42 case-control studies/ or cohort studies/ or longitudinal studies/ or follow-up studies/ or prospective studies/ or cross-sectional studies/ (1317381)

43 ((systematic adj2 review) or meta-analysis).ti. or Meta-Analysis.pt. or overview.ti. or (literature adj2 review).ti. (134074)

44 or/28,30,33 [Sets 1-3 combine with filters/study designs] (8025)

45 (44 and 36) not 43 [RCT sets 1-3] (759)

46 (44 and (37 not 35)) not (or/43,45) [Multicentre Sets 1-3] (353)

47 (44 and (38 not 35)) not (or/43,45-46) [Research Support pub types Sets 1-3] (1069)

48 (44 and (40 not 35)) not (or/43,45-47) [Comparative/Evaulation/Retro Sets 1-3] (590)

49 (44 and (42 not 35)) not (or/43,45-48) [Longitudinal, Cohort, Cross Sectional Sets 1-3] (269)

50 (44 and (41 not 35)) not (or/43,45-49) [Case Reports Sets 1-3] (366)

51 (44 and (39 not 35)) not (or/43,45-50) [Congresses Sets 1-3] (14)

52 (and/43-44) not (or/45-51) [Reveiws] (94)

53 (2003$ or 2004$ or 2005$ or 2006$ or 2007$ or 2008$ or 2009$ or 2010$ or 2011$ or 2012$ or 2013$).ed,ep,yr. (10693386)

54 (comment or editorial or letter).pt. or placebo?.ti,ab,hw. (1514226)

55 45 or 46 or 47 or 48 or 49 or 50 or 51 or 52 (3514)

56 (55 and 53) not 54 [Results 2003 forward excluding pub types requested by authors] (2338)

57 55 and 2013$.ed,ep,yr. [Jan 1-2014 update results] (295)

58 ("20661930" or "21821515" or "19342525" or "20142403").ui. [exemplar studies suggested by peer reviewers] (4)

59 ((1 and (or/36-38)) not (or/35,56)) and 53 [MedErrors & Filters new terms Jan 2014] (1223)

60 (2 not (or/35,56,59)) and 53 [Results incident reporting title screen all-Jan 2014] (185)

| **EMBASE Strategy** |
| --- |

1 medical device recalls/ or safety-based medical device withdrawals/ (187)

2 ((medical device? or medical product?) and (surveillance or safety or postmarket$ or post-market$ or after market or premarket or pre-market)).ti. (286)

3 (((cardiovascular$ or high risk or Class 3 or "class III" or implant$) adj2 device?) and (surveillance or monitoring or reporting or detecting or safety or postmarket$ or post-market$ or after market)).ti. (172)

4 (device? and (surveillance or postmarket$ or post-market$ or after market or premarket or pre-market)).ti. (232)

5 ((medical device? or medical product? or implant$ device?) adj2 adverse event?).ti,ab. (49)

6 (Fault? adj4 ((new or newer or newly or medical) adj2 (device or devices or equipment or product?))).ti,ab. (9)

7 exp *devices/ and *medical error/ (313)

8 exp devices/ and postmarketing surveillance/ (1046)

9 Postmarketing surveillance/ and (device or devices).ti. (377)

10 Postmarketing surveillance/ and (device or devices).ab. (410)

11 exp devices/ and (postmarket$ or post-market$).ti,ab. (603)

12 exp *devices/ and reporting.ti. (477)

13 exp *devices/ and (surveillance adj2 (method? or process$ or program? or progammes or scheme or schemes or model?)).ti,ab. (262)

14 exp devices/ and ((adverse event? or error? or failure) adj4 (reporting or detection or detecting or identifying)).ti,ab. (1193)

15 (device? adj5 ((adverse event? or error? or failure) adj4 (reporting or detection or detecting or identifying or identification))).ti,ab. (57)

16 exp *devices/ and "safe use".ti,ab. (364)

17 "product development"/ and *feedback system/ (3)

18 exp *devices/ and (error? or adverse event?).ti. and (report? or reporting or identification? or identify or detect? or process or procedure or programme or programmes).ti,ab. (382)

19 exp *devices/ and *feedback system/ and (report? or reporting or identification? or identify or detect? or process or procedure or programme or programmes).ti,ab. (88)

20 exp devices/ and adverse event? reporting.ti,ab. (84)

21 or/1-20 (5235)

22 exp drug/ and (safety or efficacy).ti. (19338)

23 exp food/ (626689)

24 (child or children or infant? or neonat$ or baby or p?ediatric$ or "in vitro" or newborn?).ti. or in vitro study/ or child/ or newborn/ (2786015)

25 pediatric ward/ or pediatrician/ or exp pediatrics/ (84364)

26 placebo?.ti,ab,hw. or placebo/ (315818)

27 or/22-26 [Terms to exclude] (3658400)

28 21 not 27 (4783)

29 (2003$ or 2004$ or 2005$ or 2006$ or 2007$ or 2008$ or 2009$ or 2010$ or 2011$ or 2012$ or 2013$).em,yr. (11122438)

30 28 and 29 (3449)

31 demonstration project?.ti,ab. (2223)

32 (time points adj3 (over or multiple or three or four or five or six or seven or eight or nine or ten or eleven or twelve or month$ or hour? or day? or "more than")).ab. (12071)

33 pilot.ti. or (pilot adj (project? or study or trial)).ab. (86766)

34 (multicentre or multicenter or multi-centre or multi-center).ti. (39789)

35 random$.ti,ab. or controlled.ti. (919687)

36 (control adj3 (area or cohort? or compare? or condition or design or group? or intervention? or participant? or study)).ab. (595904)

37 *experimental design/ or *pilot study/ or quasi experimental study/ (7022)

38 ("quasi-experiment$" or quasiexperiment$ or "quasi random$" or quasirandom$ or "quasi control$" or quasicontrol$ or ((quasi$ or experimental) adj3 (method$ or study or trial or design$))).ti,ab. (114503)

39 ("time series" adj2 interrupt$).ti,ab. (1162)

40 (rat or rats or cow or cows or chicken? or horse or horses or mice or mouse or bovine or animal?).ti. (1429559)

41 (exp animals/ or exp invertebrate/ or animal experiment/ or animal model/ or animal tissue/ or animal cell/ or nonhuman/) and (human/ or normal human/ or human cell/) (15119370)

42 (exp animals/ or exp invertebrate/ or animal experiment/ or animal model/ or animal tissue/ or animal cell/ or nonhuman/) not 41 (5221479)

43 (or/31-39) not (or/40,42) [Filter to find non-rct designs] (1259586)

44 30 and 43 (353)

45 controlled clinical trial/ or controlled study/ or randomized controlled trial/ [EM] (4282465)

46 randomi?ed.ti. or ((random$ or control) adj3 (group? or cohort? or patient? or hospital$ or department?)).ab. or (controlled adj2 (study or trial)).ti. (675203)

47 (random sampl$ or random digit$ or random effect$ or random survey or random regression).ti,ab. not randomized controlled trial/ [Per BMJ Clinical Evidence filter] (53480)

48 (exp animals/ or exp invertebrate/ or animal experiment/ or animal model/ or animal tissue/ or animal cell/ or nonhuman/) and (human/ or normal human/ or human cell/) (15119370)

49 (exp animals/ or exp invertebrate/ or animal experiment/ or animal model/ or animal tissue/ or animal cell/ or nonhuman/) not 48 (5221479)

50 (or/45-46) not (or/47,49) [RCT Filter for EMBASE] (2899361)

51 30 and 50 (410)

52 or/44,51 (616)

53 ((or/4,9) not (or/27,49,52)) and 29 [Unfiltered results from "high value" search concepts] (285)

54 *medical error/ (6707)

55 (report$ or prevent$ or cause or causes or causal or strateg$).ti,ab. (5261297)

56 54 and 55 (2353)

57 incident reporting.ti. (330)

58 (((or/56-57) and (or/43,50)) not (or/40,42)) and 29 [Terms added Jan 2014] (263)

59 (52 or 53) and 2013$.em,yr. [Jan 2014 update results] (161)[Original Strategy]

60 58 not 59 [New terms results Jan 2014] (262) [New Strategy Dec 2013]

| **OTHER DATABASES** | | | | | |
| --- | --- | --- | --- | --- | --- |
| PubMed | | The PubMed strategy was a focussed, truncated search on the most useful concept, namely, medical device recalls. | |  | |
| Cochrane Library, all sections  Issue 4, 2013 | | Same MeSH, keywords, as per MEDLINE search, excluding study types and Human restrictions. Syntax adjusted for Cochrane Library databases. | |  | |
| PsycINFO | | 1806 to December Week 4 2013 | |  | |
|  | | Cochrane Library (Wiley) ID Search Hits  1 ((prosthes$ or ventilat$ or device or catheter?) adj3 infection?).ti,ab,tw,kw. (720)  2 (Catheteri?ation or (Intubation adj Intratracheal) or (Ventilator? adj2 Mechanical) or (Device? adj2 Remov$) or (ventilator? adj2 Wean$) or Catheter?).ti,ab,tw,kw. (9980)  3 (((mechanical or device or artificial or assist$ or wean$) adj2 ventilat$) or (artificial adj respirat$)).ti,ab,tw,kw. (3392)  4 indwelling device?.ti,ab,tw,kw. (4)  5 or/2-4 [Devices] (12996)  6 (sepsis or septic?em$ or bacteremia or fungemia or nosocomial$ or Hospital acquired or (equipment adj2 contamination) or infection? or (ventilator? adj2 pneumonia)).ti,ab,tw,kw. (36029)  7 ((pathway? or protocol? or algorithm?) adj2 (clinical or treatment? or diagnos$ or management or infection? or infectious? or antibiotic?)).ti,ab,tw,kw. (4019)  8 critical pathway?.ti,ab. (33)  9 guidance.ti,ab. (1563)  10 (quality adj2 (improv$ or manag$ or care or healthcare)).ti,ab. (6708)  11 (guideline? adj4 (adher$ or antibiotic? or applicat$ or apply$ or clinical or complian$ or concord$ or deploy$ or diagnos$ or effect$ or efficacy or evidence or experiment$ or impact or implement$ or infectious? or infection? or introduc$ or management or pilot$ or study or treatment? or trial? or utili?ation or utili?ing or utili?e?)).ti,ab,kw,tw. (3655)  12 (protocol? adj3 (adher$ or antibiotic? or applicat$ or apply$ or clinical or complian$ or concord$ or deploy$ or diagnos$ or effect$ or efficacy or evidence or experiment$ or impact or implement$ or infectious? or infection? or introduc$ or management or pilot$ or study or treatment? or trial? or utili?ation or utili?ing or utili?e?)).ti,ab,tw,hw. (10615)  13 (pathway? or guidance or algorithm? or (quality adj2 (improv$ or manag$ or care or healthcare))).ti,ab,tw,kw. (15529)  14 or/7-13 [GL] (28465)  15 and/5-6,14 (172)  16 medical errors.mp. [mp=ti, ot, ab, sh, hw, kw, tx] (105)  17 "2013".yr. and 15 (5)  18 ("2003" or "2004" or "2005" or "2006" or "2007" or "2008" or "2009" or 201$).yr. and 16 [New terms added Jan 2014] (93)  19 or/17-18 (98)  20 from 19 keep 1-76 (76) [Cochrane Central Database of Controlled Trials]  21 from 19 keep 77-93 (17) [CDSR]  22 from 19 keep 77-92 (16)[HTA]  23 from 19 keep 93-98 (6) [EED] PubMed Search Query Items found  #11 Search "Root Cause Analysis"[Mesh] 66  #17 Search "Medical Device Recalls"[Mesh] OR ( "medical device" AND faulty) OR ("medical device" AND error) OR ("medical device" AND recall) 147 *[Original Dec 2013 strategy]*  #20 Search "Medical Errors"[Majr] 23687  #21 Search ("report"[tiab] or "reporting"[tiab] or prevent*[tiab] or cause[tiab] or causes[tiab] or causal[tiab] or strateg*[tiab]) 3307433  #22 Search (#20 AND (#21 OR #11)) 7162  #23 Search (#20 AND (#21 OR #11)) Filters: Clinical Trial 276 PSYCINFO Search Query Items found  1 incident reporting.ti. (31)  2 medical error?.ti. (96)  3 or/1-2 [Set 2] (127) | |  | |
| **Grey Literature** | | | | |  |
| Dates for Search: | | *Feb 6th and 8th 2013* | |  |  |
| Keywords: | | *device or devices AND surveillance (e.g. on medical device sites) or postmarket* post-market AND device AND safety.*. | |  |  |
| Limits: | | Publication years 1996-present | |  |  |

The following sections of the CADTH grey literature checklist, “Grey matters: a practical tool for evidence-based searching” (<http://www.cadth.ca/resources/grey-matters>) were searched:

- Health Technology Assessment Agencies
- Databases (free)
