# Supplementary material for: Factors that influence the recognition, reporting and resolution of incidents related to medical devices and other healthcare technologies: a systematic review
Source: Syst Rev. 2015 Mar 29;4:37. doi: 10.1186/s13643-015-0028-0 (PMC4384231; doi:10.1186/s13643-015-0028-0)
Supplement: Additional file 2: Table S2. — Study findings on factors that influence the recognition, reporting and resolution of incidents by healthcare professionals. The additional file presents the study findings on factors that influence the recognition, reporting and resolution of incidents by health care professionals. [file 13643_2015_28_MOESM2_ESM.docx]

Additional file 2: Table S2. **Study findings on factors that influence the recognition, reporting and resolution of incidents by health care professionals**

| **First author** | **Outcome measurement** | **Main study findings (verbatim)** | | | | | | | | | | | | | | | | | | |
| --- | --- | --- | --- | --- | --- | --- | --- | --- | --- | --- | --- | --- | --- | --- | --- | --- | --- | --- | --- | --- |
| Wong[24] | Change in clinical practice as a result of device incident reported | Device-Related Incident | | Description | | | | | | | | | Change in Practice | | | | | | | |
|  |  | Hypotony (vision loss from intraocular pressure) | | Disconnection of air tubing from machine causing hypotony due to  loose connection | | | | | | | | | Theatre staff specifically trained to check connections more carefully | | | | | | | |
|  |  | Hypotony | | Hypotony on initiation of vitrectomy: vitrector found to be connected to  wrong (pneumatic scissors) port on machine | | | | | | | | | Stopper installed at unused pneumatic scissors port | | | | | | | |
|  |  | Device: instrumentation disconnection | | High pressure cannula disconnection from syringe during injection of  viscoelastic into anterior chamber resulting in vitreous haemorrhage | | | | | | | | | Syringes with leur lock tips introduced to ensure cannula firmly  locked to syringe during use | | | | | | | |
|  |  | Device: foot pedal failure | | During pars plana vitrectomy, failure of foot pedal occurred resulting in  vitreous incarceration and iatrogenic retinal break | | | | | | | | | Foot pedals specifically checked at the start of each operating list | | | | | | | |
|  |  | Device: incorrectt microscrope filter | | During endolaser, the surgeon noticed excessive backscatter, attributed  to the placement of an incorrect filter for the wavelength of laser being used | | | | | | | | | Clearer labelling of laser and filters implemented | | | | | | | |
|  |  | Misplaced infusion port | | During vitrectomy procedure, pars plana infusion port placed in  choroidal space, instead of in vitreous cavity | | | | | | | | | Weekly consultant-led teaching session for Fellows and Residents  introduced | | | | | | | |
| Anderson[25] | Challenges faced by hospital staff in implementing an effective incident reporting system | - Acceptance of incident reporting and blame: lack of willingness or experience using system; fair blame was necessary, balance the need for accountability and a no blame culture was sometimes a challenge. - Investigation of incidents: lack of dedicated time and resources; difficulties in identifying the causes of incidents and in determining the appropriate actions to prevent a similar of occurring again. - Implementation of changes: poor quality of recommendations made in investigation reports. - Evaluation of changes: reliance mainly on informal methods of evaluation, such as team discussions, management oversight and spot checks; formal methods, such as audits and scorecards. - Feedback to staff: feedback about incident reports and related outcomes; lack of knowledge on how the system operated; difficulties of communicating this information in an effective way. | | | | | | | | | | | | | | | | | | |
| Hartnell[33] | Organizational culture or readiness for change to improve incident reporting | Organizational factors identified as barriers to medical error reporting:   - Ineffective reporting system - Lack of trust about how error reports will be used - Reporting is the responsibility of someone else | | | | | | | | | | | | | | | | | | |
|  | Incentives such as mandatory reporting or financial incentives/accountability agreements | Incentives for medical error reporting:  Patient protection   - Improved care/improved patient safety - To prevent patient from receiving wrong medication   Provider protection   - Provides immunity/protection from legal action - Fear of censure (harsh criticism or blame)   Professional compliance   - Perceived severity of error (more severe errors are more likely to be reported because a report will be expected) - Follow the rules or policies - Ensures accountability | | | | | | | | | | | | | | | | | | |
| Heard[29] | Attitudes and emotional factors influencing reporting an unspecified adverse event caused by an error | Barriers (n=430 respondents):  There were no significant differences between Error and No Error groups in terms of agreeing or strongly agreeing:  Doctors who make errors are blamed by their colleagues:  46% (CI: 42%-51%): agreed or strongly agreed  26% (CI: 22%-30%): disagreed or strongly disagreed  27% (CI: 23%-32%): neutral  Participants in the Error group were more likely to agree or strongly agree with the following barriers versus the No Error group:   - I am worried about litigation - I don’t want to get into trouble - My colleagues may be unsupportive - I am worried about disciplinary action - I may be blamed unfairly for the event - I do not want the case discussed in meetings   There were no significant differences between Error and No Error groups in terms of disagreeing or strongly disagreeing:  Adverse even reporting makes little contribution to quality of care:  93% (CI: 90%-95%): disagreed or strongly disagreed  I don’t know whose responsibility it is to make a report:  86% (CI: 83%-90%): disagreed or strongly disagreed  A good outcome of the case makes reporting unnecessary:  86% (CI: 83%-90%): disagreed or strongly disagreed  I don’t know which adverse events should be reported:  73% (CI: 68%-77%): disagreed or strongly disagreed  More than 75% (lower confidence limit) of respondents agreed or strongly agreed with 7 assistive strategies about:   - Feedback - Role models - Legislated protection - Ability to report anonymously - Clear guidelines   The majority of respondents disagreed or strongly agreed with the following strategy: ‘Payment for time taken to report’ | | | | | | | | | | | | | | | | | | |
| Flotta[36] | When a medical error occurs, physicians should: |  | % Agree | | | | | | | % Uncertain | | | | | | | % Disagree | | | |
|  |  | Conceal medical errors occurred during clinical management | 1.2 | | | | | | | 8.6 | | | | | | | 90.2 | | | |
|  |  | Avoid similar patients or analogous circumstance | 18.4 | | | | | | | 19.5 | | | | | | | 62.1 | | | |
|  |  | Increase information seeking to reduce recurrence of medical errors | 96.8 | | | | | | | 1.9 | | | | | | | 1.3 | | | |
|  |  | Discuss with colleagues about medical error during clinical management | 98.4 | | | | | | | 1.2 | | | | | | | 0.4 | | | |
|  |  | Report medical errors to their own institution to improve the quality of care | 87.6 | | | | | | | 13.9 | | | | | | | 1.5 | | | |
|  |  | Discuss with the involved patient about medical error occurred during clinical management | 44.5 | | | | | | | 44.1 | | | | | | | 11.4 | | | |
| Hwang[38] | Barriers in the operation of incident reporting systems | Barriers | | | | | | | | | | | | | | | | | | No. (%) |
|  |  | Mainly organizational factors | | | | | | | | | | | | | | | | | | 43 (44.8) |
|  |  | Constraints of incident reporting systems (e.g. no assurance of anonymity, no integrated , dual reporting systems, lack of system accessibility, usability problem; difficult to report multi-department involved incidents) | | | | | | | | | | | | | | | | | | 10 (10.4) |
|  |  | Weak safety culture (e.g., blame and punishment for person involved in the incident: blame for department involved in the incident) | | | | | | | | | | | | | | | | | | 6 (6.3) |
|  |  | Inter-department conflict and lack of cooperation (e.g., lack of cooperation from clinical departments unavailable department-specific incident cases; conflict due to which department are responsible for the incident) | | | | | | | | | | | | | | | | | | 6 (6.3) |
|  |  | Limited reporting (e.g., lack of reporting by the other department except nursing department; reporting only incidents due to external factors such as caregivers) | | | | | | | | | | | | | | | | | | 6 (6.3) |
|  |  | Intractable cases within time and financial constraints | | | | | | | | | | | | | | | | | | 6 (6.3) |
|  |  | Absence of fulltime patient safety officers | | | | | | | | | | | | | | | | | | 5 (5.2) |
|  |  | Delayed feedback | | | | | | | | | | | | | | | | | | 2 (2.1) |
|  |  | Absence of education and training opportunities on patient safety and incident reporting | | | | | | | | | | | | | | | | | | 2 (2.1) |
|  |  | Mainly individual factors | | | | | | | | | | | | | | | | | | 53 (55.2) |
|  |  | Low reporting rate | | | | | | | | | | | | | | | | | | 13 (13.5) |
|  |  | Middle-level managers lack of patient safety leadership (e.g., lack of awareness of the importance of patient safety incident reporting lack of knowledge and skills of patient safety and incident reporting, fear of blame) | | | | | | | | | | | | | | | | | | 10 (10.4) |
|  |  | Lack of physician’s reporting and participation | | | | | | | | | | | | | | | | | | 7 (7.3) |
|  |  | Top-level managers: lack of patient safety leadership | | | | | | | | | | | | | | | | | | 6 (6.3) |
|  |  | Staff: lack of knowledge and skill related to incident reporting (e.g., use of tools such as root cause analysis and failure mode and effect analysis what should be reported and how to report) | | | | | | | | | | | | | | | | | | 6 (6.3) |
|  |  | Lack of staff awareness of the importance of patient safety incident reporting | | | | | | | | | | | | | | | | | | 5 (5.2) |
|  |  | Late reporting | | | | | | | | | | | | | | | | | | 3 (3.1) |
|  |  | Fear of blame, stress | | | | | | | | | | | | | | | | | | 2 (2.1) |
|  |  | Insufficient knowledge and skills related to incident reporting of risk managers | | | | | | | | | | | | | | | | | | 1 (1.0) |
|  | Measures to resolve or overcome barriers | External   - Need for education and training programs provided by academic society - Enforcement of patient safety standards in the Healthcare Accreditation Program - Establishment of a national institute to support hospitals’ patient safety activity | | | | | | | | | | | | | | | | | | 3 (2.9) |
|  |  | Internal | | | | | | | | | | | | | | | | | | 101 (97.1) |
|  |  | Organizational | | | | | | | | | | | | | | | | | | 81 (77.9) |
|  |  | Introducing a rewarding system (e.g., rewarding for near misses, department-level rewarding link to individual performance appraisal) | | | | | | | | | | | | | | | | | | 16 (15.4) |
|  |  | Enhancing incident reporting systems (e.g., a variety of reporting channel: computer, paper, email, telephone use of data from different sources such as patient complaints and malpractice claims improving system accessibility using various platform in computers assurance of anonymity and confidentiality, improving reporting forms integrate, unified reporting systems with existing hospital information systems) | | | | | | | | | | | | | | | | | | 12 (11.5) |
|  |  | Enhancing safety culture | | | | | | | | | | | | | | | | | | 11 (10.6) |
|  |  | Providing education and training opportunities | | | | | | | | | | | | | | | | | | 11 (10.6) |
|  |  | Improving staffing for patient safety incident reporting management | | | | | | | | | | | | | | | | | | 9 (8.7) |
|  |  | Visualizing and sharing successful results/outcomes | | | | | | | | | | | | | | | | | | 7 (6.7) |
|  |  | Promotional activities (e.g., patient safety day ceremony, poster display) | | | | | | | | | | | | | | | | | | 5 (4.8) |
|  |  | Providing feedback | | | | | | | | | | | | | | | | | | 3 (2.9) |
|  |  | Designation of patient safety facilitators at the department level | | | | | | | | | | | | | | | | | | 2 (1.9) |
|  |  | Monitoring and surveying safety culture | | | | | | | | | | | | | | | | | | 1 (1.0) |
|  |  | Monitoring and surveying staff perception of patient safety | | | | | | | | | | | | | | | | | | 1 (1.0) |
|  |  | Non-punitive policy | | | | | | | | | | | | | | | | | | 1 (1.0) |
|  |  | Hospital-wide efforts to improve patient safety | | | | | | | | | | | | | | | | | | 1 (1.0) |
|  |  | Creating a formal committee dealing with incident reports | | | | | | | | | | | | | | | | | | 1 (1.0) |
|  |  | Individual | | | | | | | | | | | | | | | | | | 20 (19.2) |
|  |  | Improving staff’s awareness of patient safety and incident reporting | | | | | | | | | | | | | | | | | | 9 (8.7) |
|  |  | Strengthening patient safety leadership from top-level managers | | | | | | | | | | | | | | | | | | 7 (6.7) |
|  |  | Strengthening patient safety leadership from middle-level managers | | | | | | | | | | | | | | | | | | 4 (3.8) |
| Bodur[40] | Organizational culture or readiness for change to improve incident reporting | Frequency of events reported received the lowest score: 15% (4SD)  Frequency of reporting a mistake but is caught and corrected before affecting the resident: 13% (2 SD)  Frequency of reporting a mistake, but has no potential to harm the resident: 14% (3 SD)  Frequency of reporting a mistake that could harm the resident, but does not: 18% (4 SD)  Outcome is lower among physicians versus nurses (p <0.05) | | | | | | | | | | | | | | | | | | |
|  | Feedback provided to health professionals on rates and types of incidents | Feedback and communication openness about error: 38% (6 SD)  Lower among staff working 50 hours or more per week and in staff working in emergency/ICU/OR (p<0.05)  Feedback given to staff about changes put into place based on event reports: 30% (4 SD)  In staff working in emergency/ICU/OR (p<0.05)  Staff are informed about errors that happen in the units: 47% (9 SD)  Approached to prevent errors from happening again are discussed: 42% (6 SD)  In staff working in emergency/ICU/OR (p<0.05) | | | | | | | | | | | | | | | | | | |
|  | Incentives such as mandatory reporting or financial incentives/accountability agreements | Non-punitive response to error: 24% (4 SD)  Staff feel like their mistakes are held against them: 14% (1 SD)  When an event is reported, it feels like the person is being written up, not the problem: 19% (1 SD)  Mistakes have led to positive changes here: 42% (11 SD)  Staff worry that mistakes they make are kept in the personnel file: 23% (4 SD) | | | | | | | | | | | | | | | | | | |
| Chien[41] | Device features that influence the identification of incidents by health care professionals | Panel module and battery are errors encountered frequently that are related to the machine. | | | | | | | | | | | | | | | | | | |
|  | Patient factors that influence the identification of incidents by health care professionals | Sensors, probe and cuff account for the most frequent number of errors and are connected usually to patients, which can be damaged due to pulling and tension. | | | | | | | | | | | | | | | | | | |
| Loren[15] | Risk managers’ and physicians’ general attitudes about patient safety and error reporting |  | | | | | | | Risk Managers (n=1,472) % | | | | | Medical physicians (n=1,311) % | | | | | | P-value |
|  |  | Medical errors are one of most serious problems in health care (agree) | | | | | | | 83 | | | | | 65 | | | | | | <0.001 |
|  |  | Medical errors are usually caused by system failures (agree) | | | | | | | 84 | | | | | 58 | | | | | | <0.001 |
|  |  | Does you hospital/health care organization have an error reporting system for physicians to use to improve patient safety? | | | | | | | | | | | | | | | | | | |
|  |  | Yes | | | | | | | 15 | | | | | 16 | | | | | | 0.370 |
|  |  | No | | | | | | | 81 | | | | | 39 | | | | | | <0.001 |
|  |  | Don’t know | | | | | | | 4 | | | | | 45 | | | | | | <0.001 |
|  |  | Current systems for physicians to report patient safety problems are adequate (yes) | | | | | | | 57 | | | | | 29 | | | | | | <0.001 |
|  |  | At my hospital or healthcare organization, system changes to improve patient safety occur after errors are reported (agree) | | | | | | | 94 | | | | | 75 | | | | | | <0.001 |
|  |  | Current mechanisms to inform physicians about errors that occur in their hospitals or health care organizations are adequate (agree) | | | | | | | 51 | | | | | 17 | | | | | | <0.001 |
|  | Risk managers’ and physicians’ attitudes about error disclosure | Near misses should be disclosed (agree/strongly agree) | | | | | | | 19 | | | | | 32 | | | | | | <0.001 |
|  |  | Minor errors should be disclosed (agree/strongly agree) | | | | | | | 75 | | | | | 77 | | | | | | 0.118 |
|  |  | Serious errors should be disclosed (agree/strongly agree) | | | | | | | 98 | | | | | 98 | | | | | |  |
|  |  | Serious errors should be disclosed (strongly agree) | | | | | | | 70 | | | | | 49 | | | | | | <0.001 |
|  |  | Physicians are opposed to disclosing serious errors to patients (agree/strongly agree) | | | | | | | 39 | | | | | NA | | | | | | NA |
|  |  | Physicians are opposed to disclosing serious errors to patients (agree) | | | | | | | 73 | | | | | NA | | | | | | NA |
|  |  | Disclosure would make it LESS likely that the patient would sue (agree/strongly agree) | | | | | | | 58 | | | | | 69 | | | | | | <0.001 |
|  | Risk managers’ and physicians’ perceived barriers to disclosure (LESS likely to recommend disclosure (RM) or disclose (MD) a serious error to a patient (yes)) | Patient unaware of the error | | | | | | | 9 | | | | | 24 | | | | | | <0.001 |
|  |  | If I think the patient would not want to know about the error | | | | | | | 19 | | | | | 32 | | | | | | <0.001 |
|  |  | If I think the patient would become angry | | | | | | | 2 | | | | | 13 | | | | | | <0.001 |
|  |  | If the physician did not know the patient very well | | | | | | | 1 | | | | | 20 | | | | | | <0.001 |
|  |  | If I think the physician might get sued | | | | | | | 3 | | | | | 27 | | | | | | <0.001 |
|  |  | If I think the patient would not understand the information | | | | | | | 47 | | | | | 61 | | | | | | <0.001 |
| Malik[42] |  |  | | | | Doctors N=114  (%) | | | | | Nurses  N=103  (%) | | | | OR  (95% CI) | | | | | P-value |
|  | Reason to report: | To get immediate help for patient | | | | 25 (22) | | | | | 25 (24) | | | | 0.87 (0.46, 1.64) | | | | | 0.68 |
|  |  | To learn from mistakes | | | | 48 (42.1) | | | | | 13 (12.6) | | | | 5.035 (2.52, 10.04) | | | | | <0.001 |
|  |  | To develop a system to minimize repetition of incident | | | | 91 (79.8) | | | | | 87 (84.4) | | | | 0.727 (0.36, 1.46) | | | | | 0.37 |
|  | Reporting would be easy if reports were made to: | Colleague | | | | 22 (19.2) | | | | | 15 (14.5) | | | | 1.402 (0.68, 2.87) | | | | | 0.35 |
|  |  | Senior faculty member | | | | 20 (17.5) | | | | | 17 (16.5) | | | | 1.076 (0.52, 2.18) | | | | | 0.84 |
|  |  | Head of the department | | | | 69 (60.5) | | | | | 83 (80.5) | | | | 0.37 (0.19, 0.68) | | | | | 0.001 |
|  |  | Administration | | | | 22 (19.2) | | | | | 10 (9.7) | | | | 2.22 (0.99, 4.95) | | | | | 0.04 |
|  | Barriers to incident reporting: | Non-supportive environment, culture of shame and blame | | | | 26 (22.8) | | | | | 12 (11.6) | | | | 2.24 (1.06, 4.71) | | | | | 0.03 |
|  |  | Loss of prestige among colleagues | | | | 18 (15.7) | | | | | 9 (8.7) | | | | 1.95 (0.83, 4.57) | | | | | 0.11 |
|  |  | Legal and financial penalties | | | | 28 (24.5) | | | | | 27 (26.2) | | | | 0.91 (0.49, 1.69) | | | | | 0.77 |
|  |  | Administrative sanctions | | | | 79 (69.2) | | | | | 70 (67.9) | | | | 1.06 (0.59, 1.88) | | | | | 0.84 |
|  |  | Lack of feedback | | | | 101 (8.5) | | | | | 87 (84.4) | | | | 1.42 (0.65, 3.13) | | | | | 0.37 |
| Smits[44] | Potential prevention strategies for AEs | Percentage (%) of physicians who selected the following prevention strategies for all preventable AEs:  Quality assurance/peer review (continuously monitoring of data quality based on prespecified standards and assessment of a health professional’s performance by one or more individuals in the same field): 65%  Evaluation (evaluating the current way of behaving regarding safety): 53%  Training (improving (re)training programmes for skills needed): 50%  Procedures (completing or improving formal or informal procedures): 40%  Percentage (%) of physicians who selected the following prevention strategies for AEs with human-related causes:  Quality assurance/peer review: 68%  Percentage (%) of physicians who selected the following prevention strategies for AEs with patient-related causes:  Quality assurance/peer review: 78%  Percentage (%) of physicians who selected the following prevention strategies for AEs caused by organizational factors:  Procedures: 67% | | | | | | | | | | | | | | | | | | |
| Espin[35] | Error perception | - Deviation from standards of practice - Breaks/breaches in protocol - Negative outcomes to the patient - Repeated offenses - Scope of practice | | | | | | | | | | | | | | | | | | |
|  | Formal reporting | - Patient negligence - Actual and potential harm - Advocacy - Learning opportunities | | | | | | | | | | | | | | | | | | |
|  | Informal reporting | - Patient care issues - Harm and learning experience - Means to clarify or validate their opinions or concerns regarding patient care with managers and colleagues | | | | | | | | | | | | | | | | | | |
|  | Non-reporting | - Lack of harm to the patient - Lack of time - Fear of reprisal - Lack of management response in justifying their choices to not report | | | | | | | | | | | | | | | | | | |
| Henneman[16] | Strategies to identify errors | - Knowing all aspects of the patient - Knowing other patients in the unit - Knowing the plan of care or recognizing the lack of a comprehensive plan - Systematically assessing patients by scanning the patient and the patient’s immediate environment - Awareness of the unit’s policies and procedures - Double-checking physicians’ orders or interventions with other nurses - Using policy- and unit-based system processes - Questioning novice nurses or physicians during change-of-shift report or multidisciplinary rounds to prompt them to discuss their decision or actions in more detail | | | | | | | | | | | | | | | | | | |
|  | Strategies to interrupt errors | - Offering assistance in a nonthreatening and supportive manner - Clarifying the plan of care or the appropriateness of a plan of care | | | | | | | | | | | | | | | | | | |
|  | Strategies to correct errors | - Persevering which often involved phone calls or pages to the patient’s physician - Being physically present - Knowing the plan of care and its rationale - Offering options enabled nurses to care for patients without embarrassing or disrespecting another team member - Refer to critical care policies, hospital accreditation agencies and unit-based or other standards - Asking senior clinicians for assistance or clarification | | | | | | | | | | | | | | | | | | |
| Albolino[37] | Personal experience with patient safety issue | - Almost 90% of workers who participated in a formal incident investigation agreed on the effectiveness of this activity with respect to a positive change on patient safety. - 10% of interviewees had never been informed of a patient safety incident that occurred to a colleague. - 90% declared that they received information at least once in their careers. | | | | | | | | | | | | | | | | | | |
|  | Barriers to reporting in case of never reporting of adverse events |  | | | | | | | | | Hospital setting without IRS (n=215) | | | | | | | Hospital setting with IRS (n=160) | | |
|  |  | Fear of mistrust of colleagues | | | | | | | | | 14.4% | | | | | | | 5.6% | | |
|  |  | It is not considered a priority | | | | | | | | | 12.6% | | | | | | | 11.9% | | |
|  |  | Fear of punishment | | | | | | | | | 4.2% | | | | | | | 4.4% | | |
|  |  | Does not help to improve safety | | | | | | | | | 4.2% | | | | | | | 2.5% | | |
|  |  | Lack of time | | | | | | | | | 2.8% | | | | | | | 2.5% | | |
| Kreckler [26] | Familiarity with reporting, reporting behaviour and perceived obstacles to reporting | 1 nurse and 6 doctors did not know of local reporting system  Nurses were significantly more likely than doctors to know where to find a form: (98% vs. 43%, p<0.001)  Doctors were significantly less likely to have ever completed a form: (15% vs. 65%, p<0.001)  Nurses who completed between 2 and 4 incidents reports in the last year: 45%  Doctors who did not complete any incident reports: 69%  Factors that would significantly affect the likelihood of reporting:  Level of harm (F(1.8, 246) = 254.2, p<0.001)  Incident type (F(1.9, 258) = 64.4, p<0.001)  Profession (F(1, 135) = 20.7, p<0.001)  Nurses were almost more 3 times as likely to always report no-harm events vs. doctors  Surgical complications were far less likely to be reported than the fall, allergy and group and save incidents, regardless of profession  Lack of time, feedback, and understanding about what constitutes a patient safety incident were important barriers compared with fear, regardless of profession | | | | | | | | | | | | | | | | | | |
| Kroll[27] | A norm of selective disclosure | - Majority of participants considered their mistakes to have been minor, but there were reports of some serious errors - Most participants said they had discussed their errors informally with team members - 5 accounts described disclosure and apology to a patient - Formal reporting appeared to be selective | | | | | | | | | | | | | | | | | | |
|  | Effects of the team | - Many interviewees, especially those in ‘medical’ jobs, reported receiving ‘good’ practice supervision and support from seniors. - Juniors recognized that colleagues were crucial in preventing and minimising harm | | | | | | | | | | | | | | | | | | |
|  | Individualized blame and responsibility | - Individuals expressed a range of attitudes to error, including that it: should be avoided, should be put in perspective; had limited impact and provided a useful learning experience. - There was recurrent mention of blame, either self-blame or blame directed at others. - Explanations for error were largely focused on the individual, thereby potentially impeding these trainees from seeking advice or reflecting on other possible contributory factors. | | | | | | | | | | | | | | | | | | |
|  | Learning moment | - Many potentially valuable learning opportunities were missed, either because the senior’s response was inappropriate or because juniors did not access help. - Most learning seems to have occurred where the situation was discussed and feedback was constructive and supportive. | | | | | | | | | | | | | | | | | | |
| Hohenhaus[23] | Reporting errors | - 42% indicated “nothing” happened when they had reported an error - 22% reported that they were “disciplined by a supervisor” - 20% indicated that they had never made a medical error in their entire nursing careers - 33% stated that they had been afraid to report a medical error they had made - 66% said they had not been afraid to report - 33% had been afraid to report an error made by someone else - 51% stated they might not report if there was no harm to the patient and the error was recognized quickly - 16% wrote that they would always report an error, regardless of who made or what the circumstances were - 9% reported they might not report if a physician told them not to report the error - 7% would not report if their supervisor told them not to - 99% reported they would always report an error that resulted in harm to a patient - 25% would report any error made by a novice nurse - 77% felt that they were provided with adequate resources when faced with the decision to report a medical error - 84% felt supported by other healthcare professional colleagues and administrators when faced with reporting a medical error - 77% felt that they had been provided with adequate information and training regarding the reporting of medical errors | | | | | | | | | | | | | | | | | | |
| Cooke[34] | Reasons why a respondent would not respond to an incident |  | | | | | | Quite, very or extremely important , % (n/N) | | | | | | | | Not at all important, % (n/N) | | | | |
|  |  | Lack of organizational support | | | | | | 22.9 (27/118) | | | | | | | | 43.2 (51/118) | | | | |
|  |  | I didn’t think the incident was enough to report | | | | | | 18.6 (22/118) | | | | | | | | 47.5 (56/118) | | | | |
|  |  | Concern about my reputation: I don’t want to be seen as “accident-prone” | | | | | | 15.0 (18/120) | | | | | | | | 44.2 (53/120) | | | | |
|  |  | Desire to avoid work interruption and just carry on with the job | | | | | | 15.0 (18/120) | | | | | | | | 57.5 (69/120) | | | | |
|  |  | Too busy | | | | | | 13.7 (16/117) | | | | | | | | 53.0 (62/117) | | | | |
|  |  | Because reporting an incident would be “telling on” my colleagues | | | | | | 12.7 (15/118) | | | | | | | | 50.0 (59/118) | | | | |
|  |  | Fear of discipline | | | | | | 11.7 (14/120) | | | | | | | | 48.3 (58/120) | | | | |
|  |  | Desire not to have my name on an incident report | | | | | | 10.1 (12/119) | | | | | | | | 59.7 (71/119) | | | | |
|  |  | Concerns about the reputation of the organization | | | | | | 9.2 (11/120) | | | | | | | | 64.2 (721/120) | | | | |
|  |  | Avoidance of “red tape” associated with incident reports and investigations | | | | | | 5.9 (7/118) | | | | | | | | 57.6 (71/118) | | | | |
| Kim[39] | Nurses’ perception of patient safety culture and cooperation climate within units and across units | Selected perceptions | | | | Mean | | | | | Strongly disagree (%) | | | | Neither (%) | | | | | Strongly agree (%) |
|  |  | Perception of overall patient safety | | | | | | | | | | | | | | | | | | |
|  |  | It is by chance that more serious errors don’t happen here | | | | 4.0 | | | | | 6.1 | | | | 11.4 | | | | | 82.5 |
|  |  | We work in “crisis mode” trying to do too much, too quickly | | | | 2.8 | | | | | 32.8 | | | | 44.3 | | | | | 22.9 |
|  |  | Patient safety is never sacrificed | | | | 3.4 | | | | | 15.6 | | | | 34.7 | | | | | 49.7 |
|  |  | We have a patient safety problem here | | | | 3.5 | | | | | 12.2 | | | | 35.4 | | | | | 52.4 |
|  |  | Our systems are good at preventing errors | | | | 3.2 | | | | | 11.7 | | | | 55.2 | | | | | 33.1 |
|  |  | Unit is doing everything possible to ensure patient safety | | | | 4.0 | | | | | 2.4 | | | | 20.0 | | | | | 77.5 |
|  |  | Teamwork within units | | | | | | | | | | | | | | | | | | |
|  |  | Staff members support one another | | | | 4.1 | | | | | 0.9.3 | | | | 16.3 | | | | | 82.6 |
|  |  | Staff members treat one another with respect | | | | 3.7 | | | | | 4.6 | | | | 33.4 | | | | | 62.1 |
|  |  | Physicians and nurses work well together as a team | | | | 3.0 | | | | | 26.7 | | | | 43.2 | | | | | 30.1 |
|  |  | When a lot of work needs to be done quickly, we work together as a team | | | | 3.8 | | | | | 4.28 | | | | 26.4 | | | | | 69.1 |
|  |  | When one gets busy, others help out | | | | 3.9 | | | | | 7.7 | | | | 15.7 | | | | | 76.6 |
|  |  | Difficult to speak to physicians when there is a problem | | | | 3.5 | | | | | 14.2 | | | | 29.5 | | | | | 56.3 |
|  |  | Hospital units | | | | | | | | | | | | | | | | | | |
|  |  | Don’t coordinate well with one another | | | | 3.4 | | | | | 10.3 | | | | 42.5 | | | | | 47.1 |
|  |  | Good cooperation among them | | | | 3.3 | | | | | 10.5 | | | | 50.7 | | | | | 38.8 |
|  |  | Often unpleasant to work with staff from other units | | | | 3.4 | | | | | 15.3 | | | | 35.3 | | | | | 49.5 |
|  |  | Work well together to provide the best care | | | | 3.3 | | | | | 8.3 | | | | 52.4 | | | | | 39.3 |
| Bognár[17] | Factor analysis of selected safety attitudes domain | Safety Climate | | | | N | | | | | % Agreed | | | | Mean (SEM) | | | | | Factor loading |
|  |  | Debriefing after errors occur is common | | | | 48 | | | | | 29 | | | | 2.92 (0.14) | | | | | 0.68 |
|  |  | The culture in our OR makes it easy to learn from mistakes of others | | | | 51 | | | | | 43 | | | | 2.68 (0.13) | | | | | 0.65 |
|  |  | I received appropriate feedback about my performance | | | | 55 | | | | | 62 | | | | 2.20 (0.13) | | | | | 0.49 |
|  |  | It is difficult to discuss mistakes when they occur in the OR | | | | 52 | | | | | 60 | | | | 2.42 (0.14) | | | | | 0.41 |
|  | Factor analysis of selected error burden domain | Error management | | | | | | | | | | | | | | | | | | |
|  |  | My department provides adequate, timely information about events in the hospital that might affect my work | | | | 59 | | | | | 68 | | | | 2.10 (0.14) | | | | | 0.82 |
|  |  | I am encouraged by my colleagues to report any patient safety concerns | | | | 59 | | | | | 78 | | | | 1.94 (0.13) | | | | | 0.74 |
|  |  | I know the proper channels to direct questions regarding patient safety in my department or work area | | | | 57 | | | | | 95 | | | | 1.51 (0.09) | | | | | 0.67 |
|  |  | Our levels of staffing are sufficient to handle the number patients | | | | 52 | | | | | 31 | | | | 2.79 (0.12) | | | | | 0.55 |
|  |  | Trainees in my discipline (e.g., nurses, residents) are adequately supervised | | | | 53 | | | | | 79 | | | | 1.83 (0.11) | | | | | 0.53 |
|  |  | I have used the hospital’s reporting system for documenting medical errors | | | | 51 | | | | | 61 | | | | 2.23 (0.19) | | | | | 0.52 |
|  |  | Decision making in our OR should include more input from others OR staff than it does now | | | | 50 | | | | | 74 | | | | 1.96 (0.11) | | | | | 0.51 |
|  |  | Disruptions in continuity of patient care can be detrimental to patient safety | | | | 58 | | | | | 93 | | | | 1.48 (0.08) | | | | | 0.48 |
|  |  | Problems with equipment are frequent in the OR | | | | 52 | | | | | 69 | | | | 2.17 (0.14) | | | | | 0.46 |
|  |  | We have a confidential reporting system for documenting medical errors | | | | 47 | | | | | 85 | | | | 1.65 (0.13) | | | | | 0.42 |
|  |  | When medical errors occur they are handled appropriately | | | | 53 | | | | | 85 | | | | 1.77 (0.11) | | | | | 0.42 |
|  |  | Risk Modification | | | | | | | | | | | | | | | | | | |
|  |  | I am properly trained to use and existing equipment in the OR | | | | 55 | | | | | 95 | | | | 1.71 (0.11) | | | | | 0.77 |
|  |  | Errors because of lack of skill are rare in the OR | | | | 52 | | | | | 58 | | | | 2.23 (0.13) | | | | | 0.75 |
|  |  | The OR equipment in our hospital is adequate | | | | 53 | | | | | 47 | | | | 2.58 (0.15) | | | | | 0.72 |
|  |  | Errors because of lack of knowledge are rare in the OR | | | | 54 | | | | | 59 | | | | 2.24 (0.13) | | | | | 0.70 |
|  |  | I would feel perfectly safe as a patient in our OR | | | | 59 | | | | | 64 | | | | 2.09 (0.14) | | | | | 0.66 |
|  |  | I am afraid to report adverse events as I might be punished or lose my job | | | | 60 | | | | | 12 | | | | 3.57 (0.10) | | | | | 0.53 |
|  |  | I am reluctant to report adverse events as I might get a colleague or friend in trouble | | | | 60 | | | | | 15 | | | | 3.45 (0.09) | | | | | 0.52 |
|  |  | I expect to be consulted on matters that affect the performance of my duties | | | | 55 | | | | | 95 | | | | 1.29 (0.08) | | | | | 0.40 |
| Evans[30] |  | Control reports | Intervention reports | | | | | | | Rate ratio (95% CI) | | | | | | | Absolute difference (SEM) | | | |
|  | Reporting rates/10,000 OBDs: ICU | 17.0 (7/4,107) | 118.2 (153/12,943) | | | | | | | 0.1 (0 to 0.3) | | | | | | | 34.0 (31.7) | | | |
|  | Reporting rates/10,000 OBDs: Surgical | 71.9 (140/19,458) | 150.8 (165/10,940) | | | | | | | 0.5 (0.4 to 0.6) | | | | | | | 76.3 (29.7) | | | |
|  | Reporting rates/10,000 OBDs: Medical | 141.0 (313/22,197) | 243.1 (612/25,178) | | | | | | | 0.6 (0.5 to 0.7) | | | | | | | 84.5 (30.4) | | | |
|  | Reporting rates/10,000 OBDs: Total inpatient reporting | 101.0 (462/45,762) | 189.6 (930/49,061) | | | | | | | 0.5 (0.5 to 0.6) | | | | | | | 60.3 (18.4) | | | |
|  | Reporting rates/10,000 ED attendance | 22.2 (86/38,760) | 46.5 (181/28,888) | | | | | | | 0.4 (0.3 to 0.5) | | | | | | | 39.5 (11.5) | | | |
|  | Reporting rates/10,000 OBDs: Doctors | 0.7 (3/45,762) | 6.3 (31/49,061) | | | | | | | 0.1 (0 to 0.3) | | | | | | | 5.2 (3.6) | | | |
|  | Reporting rates/10,000 OBDs: Nurses | 88.5 (405/45,762) | 177.3 (870/49,061) | | | | | | | 0.5 (0.4 to 0.6) | | | | | | | 59.0 (17.9) | | | |
|  | Reporting rates/10,000 ED attendances: Doctors | 0.3 (1/38,760) | 9.0 (26/28,888) | | | | | | | 0.03 (0 to 0.2) | | | | | | | 9.5 (3.7) | | | |
|  | Reporting rates/10,000 ED attendances: Nurses | 19.3 (75/38,760) | 31.2 (90/28,888) | | | | | | | 0.6 (0.5 to 0.8) | | | | | | | 24.8 (9.2) | | | |
| Evans[32] | Awareness and use of the incident reporting system | Doctors (93.6%; 174/186) vs. nurses (99.8%; 586/587): RR=1.01 (95% CI: 0.99 to 1.03) | | | | | | | | | | | | | | | | | | |
|  | Ever completed an incident report | Doctors (64.6%; 115/186) vs. nurses (89.2%; 520/587): RR=1.38 (95% CI: 1.19 to 1.61) | | | | | | | | | | | | | | | | | | |
|  | Know how to located/access an incident form | Doctors (43.0%; 77/186) vs. nurses (88.3%; 515/587): RR=2.05 (95% CI: 1.61 to 2.63) | | | | | | | | | | | | | | | | | | |
|  | Know what to do with a completed incident form | Doctors (49.7%; 89/186) vs. nurses (81.9%; 476/587): RR=1.65 (95% CI: 1.27 to 2.13) | | | | | | | | | | | | | | | | | | |
|  | Staff self-perception of reporting of incidents | Incident | | | | | Doctors (n=186; %) | | | | | Nurses (n=576; %) | | | | | | | P-value | |
|  |  | Patient falls should always be reported | | | | | 75.8 | | | | | 97.0 | | | | | | | NR | |
|  |  | Drug error near misses should ways be reported | | | | | 42.1 | | | | | 41.9 | | | | | | | NR | |
|  | Self-perceived barriers to reporting (percentage who agree with the statement) | I never get any feedback on what action is taken: | | | | | 57.7 | | | | | 61.8 | | | | | | | 0.371 | |
|  |  | The incident form takes too long to fill out and I just don’t have the time | | | | | 54.2 | | | | | 44.1 | | | | | | | 0.022 | |
|  |  | The incident was too trivial | | | | | 51.2 | | | | | 41.2 | | | | | | | 0.027 | |
|  |  | When the ward is busy I forget to make a report | | | | | 47.3 | | | | | 48.1 | | | | | | | 0.930 | |
|  |  | I don’t know whose responsibility it is to make a report | | | | | 37.9 | | | | | 10.8 | | | | | | | <0.001 | |
|  |  | When it is a near miss, I don’t see any point in reporting it | | | | | 36.0 | | | | | 49.0 | | | | | | | 0.003 | |
|  |  | The AIMS+form us too complicated and required too much detail | | | | | 31.9 | | | | | 35.0 | | | | | | | 0.512 | |
|  |  | Junior staff are often blamed unfairly | | | | | 31.0 | | | | | 25.6 | | | | | | | 0.169 | |
|  |  | Adverse incident reporting is unlikely to lead to system changes | | | | | 28.6 | | | | | 29.9 | | | | | | | 0.775 | |
|  |  | I wonder about who else is privy to the information that I disclose | | | | | 27.1 | | | | | 33.8 | | | | | | | 0.112 | |
|  |  | If I discuss the case with the person involved nothing else needs to be done | | | | | 24.9 | | | | | 11.5 | | | | | | | <0.001 | |
|  |  | I don’t feel confident the form is kept anonymous | | | | | 22.6 | | | | | 30.0 | | | | | | | 0.065 | |
|  |  | I am worried about litigation | | | | | 20.7 | | | | | 20.6 | | | | | | | 1.000 | |
|  |  | It’s not my responsibility to report somebody else’s mistakes | | | | | 17.2 | | | | | 16.4 | | | | | | | 0.814 | |
|  |  | My co-workers may be unsupportive | | | | | 13.8 | | | | | 20.8 | | | | | | | 0.045 | |
|  |  | I don’t want to get into trouble | | | | | 10.6 | | | | | 18.6 | | | | | | | 0.014 | |
|  |  | Even if I don’t give my details, I’m sure that they’ll track me down | | | | | 8.4 | | | | | 17.0 | | | | | | | 0.006 | |
|  |  | I am worried about disciplinary action | | | | | 8.3 | | | | | 18.1 | | | | | | | 0.002 | |
|  |  | I don’t want the case discussed in meetings | | | | | 7.2 | | | | | 15.5 | | | | | | | 0.005 | |
| Schectman[18] | Reporting behaviour | - 49 physicians (41% of 120 respondents) were not familiar with the safety process - 12 physicians (10% of 120 respondents) were very familiar with the safety process - 39 physicians (33% of 119 respondents) knew how to report an AE or a near miss - 55 physicians (46% of 120 respondents) were aware of QI initiatives at the hospital that were based on the safety and quality monitoring systems | | | | | | | | | | | | | | | | | | |
|  | Barriers | Reason | | | | | Not Important | | | | | Somewhat Important | | | | | | | Very Important | |
|  |  | Unsure of reporting mechanism | | | | | 29% (35) | | | | | 41% (49) | | | | | | | 29% (35) | |
|  |  | No actual harm came to patient | | | | | 32% (38) | | | | | 45% (54) | | | | | | | 23% (27) | |
|  |  | Reporting too difficult or time-consuming | | | | | 32% (38) | | | | | 42% (49) | | | | | | | 26% (31) | |
|  |  | Unsure whose responsibility to report | | | | | 34% (41) | | | | | 48% (57) | | | | | | | 18% (21) | |
|  |  | Unsure of what is considered AE/NM | | | | | 36% (43) | | | | | 48% (57) | | | | | | | 16% (19) | |
|  |  | Inadequate M.D. participation in system | | | | | 36% (43) | | | | | 40% (48) | | | | | | | 24% (28) | |
|  |  | Concern about consequences of reporting other’s error | | | | | 44% (52) | | | | | 42% (50) | | | | | | | 14% (16) | |
|  |  | Reporting makes no difference (nothing will change) | | | | | 45% (54) | | | | | 42% (50) | | | | | | | 13% (15) | |
|  |  | Concern about being blamed or judged less competent | | | | | 64% (76) | | | | | 26% (31) | | | | | | | 9% (11) | |
|  | Ways to increase incident reporting | Item | | | | | Unlikely | | | | | Somewhat Unlikely | | | | | | | Very Likely | |
|  |  | Allow electronic reporting of AEs/NMs | | | | | 2% (2) | | | | | 27% (32) | | | | | | | 71% (84) | |
|  |  | Clarify reporting mechanism | | | | | 5% (6) | | | | | 42% (50) | | | | | | | 53% (62) | |
|  |  | Clarify what constitutes an AE/NM | | | | | 8% (10) | | | | | 39% (46) | | | | | | | 53% (62) | |
|  |  | Allow anonymous reporting | | | | | 8% (9) | | | | | 38% (45) | | | | | | | 54% (63) | |
|  |  | Increase physician involvement in QI process | | | | | 14% (16) | | | | | 51% (59) | | | | | | | 35% (41) | |
|  |  | Provide feedback on QI projects arising from reports | | | | | 15% (17) | | | | | 55% (64) | | | | | | | 31% (36) | |
|  |  | Provide individual feedback following report | | | | | 17% (20) | | | | | 56% (66) | | | | | | | 27% (32) | |
|  |  | Provide summary feedback on a regular basis | | | | | 21% (24) | | | | | 53% (61) | | | | | | | 27% (31) | |
|  |  | Make reporting mandatory | | | | | 38% (45) | | | | | 35% (41) | | | | | | | 26% (31) | |
| Ursprung[19] | Frequency of errors detected by safety audits | - 338 errors - Most errors were detected at bedside, allowing for immediate feedback to clinical staff (data not provided) | | | | | | | | | | | | | | | | | | |
|  | Policy changes and educational initiatives resulting from information obtained via patient safety audits | - Development of a pulse oximeter saturation guideline. - Education of the clinical staff as to optimal oxygen saturation targets for various clinical conditions. - Change in the patient identification system used in the NICU. - Education of the nursing staff as to the hospital policy concerning identification bands. - Nursing leadership participation in a follow up safety audit study: revision of safety audit questions, creation of new safety audit questions; staff emails concerning findings of the study. - An intermediate care unit in the hospital learned of the audits and started their own unit based safety audit system | | | | | | | | | | | | | | | | | | |
|  | Feedback on use of patient safety audits in clinical practice | - Only one concern of auditing was reported to the research nurse or to NICU leadership. - Several clinical staff members reported that auditing 5–7 questions per patient during the work rounds was time consuming, occasionally disrupting the flow of rounds. - Many staff expressed enthusiasm for continued auditing during work rounds provided that only one or two safety questions were addressed per patient. - Many clinicians (nurses, nurse practitioners, and physicians) expressed interest in being a ‘‘safety auditor’’. | | | | | | | | | | | | | | | | | | |
| Mazeau[43] | Feedback provided to health professionals on rates and types of incidents | Higher percentage of head nurses and nurses (100) and other caregivers (89) correctly knew whom to alert if faced with a hospital incident versus physicians (74) and administrative staff (82) (p<0.001)  No statistically significant difference among physicians (39), head nurses and nurses (59), other caregivers (54) and administrative staff (35) in correct identification of hospital device surveillance correspondent in their hospital (p=0.076) | | | | | | | | | | | | | | | | | | |
|  | Awareness of a reporting/surveillance system | Higher percentage of physicians (88), head nurses and nurses (95) correctly responded to medical device definition versus other caregivers (64) and administrative staff (65) (p<0.001)  Higher percentage of physicians (77), head nurses and nurses (69) correctly identified medical devices versus other caregivers (53) and administrative staff (41) (p=0.004)  No statistically significant difference among physicians (55), head nurses and nurses (53), other caregivers (50) and administrative staff (41) in correct identification of incidents listed in survey that concern hospital device surveillance (p=0.75)  Higher percentage of physicians (90), head nurses and nurses (95) correctly identified incidental situations that required hospital device surveillance procedure versus other caregivers (83) and administrative staff (71) (p=0.03) | | | | | | | | | | | | | | | | | | |
| Demiris[20] | System factors that influence the identification of incidents by health care professionals | Health care providers stated that an electronic reporting system would be useful and could have potential to shorten the reaction time after an event has been reported (data not provided)  Features a reporting system should have in order to be accepted and utilized by care providers:   - Easy to use - Fast - Require minimal training - Accessible - Provide statistics | | | | | | | | | | | | | | | | | | |
|  | Incentives such as mandatory reporting or financial incentives/accountability agreements | Percentage of providers believed that there was no culture of blame that was regularly placed on individuals involved in medical errors: 71% (n=10/14) | | | | | | | | | | | | | | | | | | |
|  | Education or training on how to identify/report incident and associated costs | Potential challenges associated with transition to an electronic reporting system:   - Training time and cost - Implementation and maintenance costs | | | | | | | | | | | | | | | | | | |
| Cohen[21] | Incentives such as mandatory reporting or financial incentives/accountability agreements | Results showed an increase in staff satisfaction with the safety measures in place and less fear of punishment or retribution for reporting medical error (data not reported) | | | | | | | | | | | | | | | | | | |
|  | Awareness of a reporting/surveillance system | Results demonstrated an increase in staff’s awareness of the importance of patient safety and willingness to report safety events (p<0.001) | | | | | | | | | | | | | | | | | | |
| Kingston[31] | Barriers to incident reporting | Barriers to incident reporting | | | Suggested strategies | | | | | | | | | | | | | | | |
|  |  | Lack of knowledge about the process and what constitutes an incident | | | - Education at orientation - Ongoing education and use of case studies to highlight reportable events at departmental forums - Ability to access a reference manual about what to report | | | | | | | | | | | | | | | |
|  |  | “Nursing Forum” by association | | | - Rename/redesign the form to make it more relevant to medical staff | | | | | | | | | | | | | | | |
|  |  | Time constraints and complexity of reporting form | | | - Simplify the reporting process-one page being the optimal length - Option of quicker reporting processes (e.g., telephone reporting, online submission) | | | | | | | | | | | | | | | |
|  |  | Lack of feedback when report generated | | | - Risk-assessment tool to prioritize action for follow-up - Type and timing of feedback given dependent on severity of incident or degree of risk to the organization. - Staff informed of follow-up processes | | | | | | | | | | | | | | | |
|  |  | Lack of legal privilege afforded to the reporting process | | | - Education to explain current legal privilege afforded through Australian federal legislation - Confidentiality guaranteed - Increased confidentiality and security of the reporting process to prevent access to information by unauthorized personnel | | | | | | | | | | | | | | | |
|  |  | Culture blame | | | - Option to report anonymously to an independent body, without fear of being identified and with the option to omit identifiers if either self and/or organization - Education | | | | | | | | | | | | | | | |
|  |  | No value | | | - Individual/group feedback of action taken | | | | | | | | | | | | | | | |
| Waring[28] | Cultural Barriers to Reporting | The fear of blame and the fear of reporting | | | - All doctors involved in the research made reference to the ‘blame thing’ or a ‘blame culture’ when expressing their apprehensions about incident reporting. | | | | | | | | | | | | | | | |
|  |  | The inevitability of error and the purpose of reporting | | | - This research revealed that from working on the premise of ‘perfection’ (Leapre, 1999), doctors regarded errors as an inevitable and sometimes beneficial dimension of their work. - It was found that the majority of physicians believed all human activity was prone to error. - In consequence, the doctors tended to regard incident reporting as a managerial exercise and questioned its contribution to service quality. | | | | | | | | | | | | | | | |
|  |  | Rejection of bureaucracy and managerial scrutiny | | | - A prominent theme that characterised the views of doctors and emerged from the issues raised above was a strong revulsion of what was often termed ‘bureaucracy’, ‘red tape’, ‘admin’, and management. - The doctors were particularly concerned that the growing number of bureaucratic hospital procedures would reduce their capacity for ‘real’ medical work. | | | | | | | | | | | | | | | |
|  |  | Divergent occupational responsibilities and expertise | | | - In the light of the above findings, one of the most interesting themes in the interviews was the sentiment that incident reporting was designed and operated primarily for other occupational groups that were more suited to bureaucratic procedures, especially ‘nursing’. - Doctors often claimed that incident reporting grew out of the nursing profession because its culture was familiar with ‘form filling’ and ‘paper work’ and more amenable to managerial control. | | | | | | | | | | | | | | | |
| Jeffe[22] | Barriers to Error Reporting | - Not knowing what to report - Not knowing how to report - Fear of repercussions (culture of blame) - Lack of confidentiality - Lack of time and easy systems for reporting - Lack of follow-up | | | | | | | | | | | | | | | | | | |
|  | Facilitators to Error Reporting | - Clear guidelines - Clarify reporting mechanisms and train health care providers (especially physicians) to use them - Nonaccusatory, mentoring - Nonaccusatory, mentoring/collegial environment - Anonymous reporting mechanisms - Sufficient personnel and efficient reporting tools - Routine follow-up of error reports for educational purposes and to show them that hospital will act on error reports | | | | | | | | | | | | | | | | | | |

AE: adverse event; Adverse medical device event= the adverse medical device event; AIMS=Australian Incident Monitoring System; CI=confidence interval; CUSA=cavitron ultrasonic surgical aspirator; CVA=cardiovascular accident; ED=emergency department; ICD=international classification of diseases; ICU=intensive care unit; LOS: length of stay; IRS=incident reporting system; MD=medical doctor; NICU=neonatal intensive care unit; NM=near miss; OBD=occupied day bed; OR=odds ratio; OR=operating room; PSI=patient safety incident; QI=quality improvement; RM=risk manager; RR=relative risk; SD=standard deviation; SEM=standard error of the mean; VR=vitreoretinal
